# Supplementary material for: Community and functional stability in a working bioreactor degrading 1,4-dioxane at the Lowry Landfill Superfund Site
Source: Appl Environ Microbiol. 2025 Sep 23;91(10):e00574-25. doi: 10.1128/aem.00574-25 (PMC12542641; doi:10.1128/aem.00574-25)
Supplement: Supplemental material — Tables S1 to S6; Fig. S1 to S9. [file aem.00574-25-s0003.pdf]

Supplemental Materials

Community and functional stability in a working bioreactor degrading 1,4-dioxane at the Lowry Landfill Superfund Site

Jessica L. Romero[1], Jack H. Ratliff[1], Christopher J. Carlson[2], Daniel R. Griffiths[2], Christopher S. Miller[1], Annika C. Mosier[1], Timberley M. Roane[1]*

[1] Department of Integrative Biology, University of Colorado Denver

[2] Parsons Corporation

* corresponding author: timberley.roane@ucdenver.edu

Table S1. Categories for the 39 SDIMOs described in K. L. Goff and L. A. Hug (1).

SDIMO category	Full description	Total	Sequence use in K. L. Goff and L. A. Hug (1)
CDDP	Candidate dioxane degrading protein	8	Reference proteins from the literature with evidence of dioxane degradation that were searched for in genomes of known dioxane-degrading bacteria using BLASTp and in environmental metagenomes
OUT	Outgroup protein	5	Proteins from the literature with no evidence of dioxane degradation that were used as an anchor in phylogenetic trees
COMP	Composite protein	17	BLASTp-curated proteins from the genomes of known dioxane-degrading bacteria that shared either a high (>90%) or moderate (25-90%) percent identity with a CDDP
COMPOUT	Composite outgroup protein	9	BLASTp-curated proteins from the genomes of known dioxane-degrading bacteria that shared either a high (>90%) or moderate (25-90%) percent identity with a CDDP and were eventually presumed to not degrade dioxane based on phylogenetic placement

Table S2. List of the 39 SDIMOs described in K. L. Goff and L. A. Hug (1).

	Abbreviation	Description	Accession	KO	Group	Length (amino acids)
**1**	CDDP1	*DxmA [Pseudonocardia dioxanivorans CB1190]*	WP_103383250.1	K18223	V	545
**2**	CDDP2	*PrmA [Mycobacterium dioxanotrophicus PH-06]*	WP_087083743.1	NA	VI	512
**3**	CDDP3	*TmoA [Azoarcus sp. DD4]*	QDF97112.1	K15760	II	501
**4**	CDDP4	*TomA3 [Burkholderia cepacia G4]*	AAK07411.1	K16242	I	519
**5**	CDDP5	*sMMO [Methylosinus trichosporium OB3b]*	ATQ70365.1	K16157	III	526
**6**	CDDP6	*TmoA* [Pseudomonas mendocina KR1]*	AAA25999.1	K15760	II	500
**7**	CDDP7	*TbuA1 [Ralstonia pickettii PKO1]*	AAB09618.1	K15760	II	501
**8**	CDDP8	*PrmA* [Rhodococcus sp. RR1]*	ADM83577.1	K18223	V	439
**9**	OUT1	*alpha subunit-terminal oygenase component [Pseudomonas sp.]*	AAA88459.1	K16242	I	513
**10**	OUT2	*toluene, o-xylene monooxygenase oxygenase subunit [Pseudomonas sp. OX1]*	CAA06654.1	K15760	II	498
**11**	OUT3	*Methane monooxygenase [Pseudonocardia dioxanivorans CB1190]*	AEA22892.1	K18223	V	550
**12**	OUT4	*methane monooxygenase [Pseudonocardia acaciae]*	WP_156993170.1	K18223	V	550
**13**	OUT5	*methane monooxygenase [Pseudonocardia asaccharolytica]*	WP_147201036.1	K18223	V	550
**14**	COMP1	*butane monooxygenase alpha subunit [Brachymonas petroleovorans]*	AAR98534.1	K16157	III	535

Table S2. List of the 39 SDIMOs described in K. L. Goff and L. A. Hug (1) (continued).

	Abbreviation	Description	Accession	KO	Group	Length (amino acids)
**15**	COMP2	*aromatic/alkene/methane monooxygenase hydroxylase/oxygenase subunit alpha [Methylococcus capsulatus]*	WP_010960482.1	K16157	III	527
**16**	COMP3	*MULTISPECIES: aromatic/alkene/methane monooxygenase hydroxylase/oxygenase subunit alpha [Methylosinus]*	WP_003609337.1	K16157	III	526
**17**	COMP4	*butane monooxygenase hydroxylase BMOH alpha subunit [Thauera butanivorans]*	AAM19727.1	K16157	III	530
**18**	COMP5	*aromatic/alkene/methane monooxygenase hydroxylase/oxygenase subunit alpha [Mycobacterium sp. ENV421]*	WP_102810306.1	NA	VI	513
**19**	COMP6	*MULTISPECIES: aromatic/alkene/methane monooxygenase hydroxylase/oxygenase subunit alpha [Rhodococcus]*	WP_006947300.1	NA	VI	512
**20**	COMP7	*soluble di-iron monooxygenase alpha subunit, partial [Pseudonocardia sp. D17]*	BAU36819.1	K18223	V	127
**21**	COMP8	*tetrahydrofuran monooxygenase oxygenase component alpha subunit [Pseudonocardia sp. ENV478]*	AEI99544.1	K18223	V	545

Table S2. List of the 39 SDIMOs described in K. L. Goff and L. A. Hug (1) (continued).

	Abbreviation	Description	Accession	KO	Group	Length (amino acids)
**22**	COMP9	*methane monooxygenase [Pseudonocardia sp. N23]*	WP_098956496.1	K18223	V	545
**23**	COMP10	*alpha-subunit of multicomponent tetrahydrofuran monooxygenase [Pseudonocardia tetrahydrofuranoxydans]*	CAC10506.1	K18223	V	545
**24**	COMP11	*putative alkene monooxygenase alpha subunit [Mycolicibacterium rhodesiae JS60]*	AAO48576.1	K18223	IV	500
**25**	COMP12	*methane monooxygenase [Pseudonocardia asaccharolytica]*	WP_028928894.1	K18223	V	547
**26**	COMP13	*Propane 2-monooxygenase, hydroxylase component large subunit [Rhodococcus jostii RHA1]*	Q0SJK9.1	K18223	V	544
**27**	COMP14	*propane monooxygenase hydroxylase large subunit [Gordonia sp. TY-5]*	BAD03956.2	K18223	V	545
**28**	COMP15	*methane monooxygenase [Mycobacterium goodii]*	WP_049747756.1	K18223	V	542
**29**	COMP16	*methane monooxygenase [Rhodococcus jostii]*	WP_011593714.1	K18223	V	544
**30**	COMP17	*methane monooxygenase [Mycolicibacterium smegmatis]*	WP_003893346.1	K18223	V	542

Table S2. List of the 39 SDIMOs described in K. L. Goff and L. A. Hug (1) (continued).

	**Abbreviation**	**Description**	**Accession**	**KO**	**Group**	**Length (amino acids)**
**31**	COMPOUT1	*MULTISPECIES: aromatic/alkene/methane monooxygenase hydroxylase/oxygenase subunit alpha [Acinetobacter]*	WP_033917438.1	K16242	I	558
**32**	COMPOUT2	*MULTISPECIES: aromatic/alkene/methane monooxygenase hydroxylase/oxygenase subunit alpha [Burkholderia cepacia complex]*	WP_060217674.1	K16242	I	513
**33**	COMPOUT3	*phenol 2-monooxygenase [Ralstonia pickettii DTP0602]*	AGW89993.1	K16242	I	503
**34**	COMPOUT4	*Tbc1D monooxygenase [Burkholderia cepacia]*	AAG40791.1	K16242	I	514
**35**	COMPOUT5	*aromatic/alkene/methane monooxygenase hydroxylase/oxygenase subunit alpha [Ralstonia pickettii]*	WP_012430588.1	K16242	I	514
**36**	COMPOUT6	*putative isoprene monooxygenase alpha subunit [Rhodococcus sp. AD45]*	CAB55825.1	K15760	II	514
**37**	COMPOUT7	*alkene monooxygenase system oxygenase component subunit alpha [Xanthobacter autotrophicus]*	WP_011992972.1	K15760	II	497
**38**	COMPOUT8	*DMS oxygenase component [Acinetobacter sp.]*	BAA23333.1	K16242	I	511
**39**	COMPOUT9	*Phenol 2-monooxygenase, oxygenase component DmpN [Pseudomonas sp. CF600]*	P19732.1	K16242	I	517

Table S3. Lowry Landfill Bioreactor 1 support media 16S rRNA sample (n=23) read counts and alpha diversity values.

	Read Count	Observed	Shannon	Simpson	Faith PD
19-03-26-R1-N1-A	66685	393	4.2	0.06	28
19-03-26-R1-N1-B	134655	508	4.2	0.04	36
19-03-26-R1-N1-C	100060	471	4.2	0.04	33
19-03-26-R1-N1-D	13456	204	4.1	0.12	19
19-03-26-R1-N2-C	48664	399	4.4	0.06	31
22-01-18-R1-N1-B	12780	210	4.3	0.14	18
22-01-18-R1-N1-C	5336	113	3.9	0.20	11
22-01-18-R1-N1-D	16537	257	4.5	0.14	20
22-01-18-R1-N2-A	23076	282	4.3	0.09	23
22-01-18-R1-N2-B	8653	160	4.1	0.16	14
22-01-18-R1-N2-C	7449	142	4.0	0.17	14
22-01-18-R1-N2-D	26756	318	4.5	0.10	24
22-01-25-R1-N1-A	28522	259	4.2	0.09	19
22-01-25-R1-N1-B	13266	179	4.1	0.14	15
22-01-25-R1-N1-D	10860	190	4.2	0.16	17
22-01-25-R1-N2-A	10688	177	4.0	0.12	14
22-01-25-R1-N2-B	7491	125	3.8	0.15	12
22-01-25-R1-N2-D	19469	231	4.1	0.09	19
22-03-22-R1-N1-B	14870	211	4.2	0.12	18
22-03-22-R1-N1-C	6574	135	4.0	0.20	12
22-03-22-R1-N1-D	10211	167	4.1	0.16	17
22-03-22-R1-N2-B	10543	191	4.3	0.18	17
22-03-22-R1-N2-D	13121	212	4.3	0.16	18

Table S4. Lowry Landfill Bioreactor 1 support media metagenomic shotgun sequencing sample (n=12) information and assembly statistics.

Sample Name	Date	Number of Contigs	Number of Contigs Greater Than or Equal to 1000 bp	N50	Smallest Contig	Largest Contig	Number of Proteins
**S1**	2019-03-26	1870969	313996	3044	250	1010632	3151125
**S2**	2019-03-26	2119006	371005	3408	250	1073174	3645417
**S3**	2019-03-26	1900365	313886	3386	250	1010632	3240958
**S4**	2022-01-18	2260283	347124	2999	250	1010632	3761288
**S5**	2022-01-18	2837071	429127	2622	250	1010632	4575244
**S6**	2022-01-18	2171795	344778	3058	250	767858	3628060
**S7**	2022-01-25	2399178	359486	2839	250	1010632	3935211
**S8**	2022-01-25	2272037	372938	2845	250	1010632	3773237
**S9**	2022-01-25	2438870	412304	3268	250	1190090	4160762
**S10**	2022-03-22	2527501	407399	3009	250	1010632	4212789
**S11**	2022-03-22	2652858	430838	3383	250	1010632	4521304
**S12**	2022-03-22	2626798	411297	3076	250	1010632	4364332

	Query (1,4-dioxane degradation pathway enzyme proteins)	Accession	Paper	Strain	Function	Pathway Enzyme	KO	Definition
1	Psed_6977 | Ferredoxin--NAD(+) reductase (plasmid) [Pseudonocardia dioxanivorans CB1190]	AEA29038	Grostern	CB1190	upregulated with dioxane	monooxygenase	K18225	prmB; propane monooxygenase reductase component [EC:1.18.1.-]
2	Psed_6978 | methane/phenol/toluene hydroxylase (plasmid) [Pseudonocardia dioxanivorans CB1190]	AEA29039	Grostern	CB1190	upregulated with dioxane	monooxygenase	K18224	prmC; propane 2-monooxygenase small subunit [EC:1.14.13.227]
3	Psed_6979 | monooxygenase component MmoB/DmpM (plasmid) [Pseudonocardia dioxanivorans CB1190]	AEA29040	Grostern	CB1190	upregulated with dioxane	monooxygenase		
4	YHS protein		Chen	Rhizobiales	initial hydroxylation	monooxygenase	K15760	tmoA, tbuA1, touA; toluene monooxygenase system protein A [EC:1.14.13.236 1.14.13.-]
5	4-Hydroxyphenylacetate 3-hydroxylase		Chen	Xanthobacter	initial hydroxylation	monooxygenase		
6	putative ferredoxin NAD(+) reductase [Bradyrhizobium sp. BTAi1]	ABQ33807	Dai	Ancylobacter polymorphus ZM13	initial hydroxylation	monooxygenase		
7	toluene 4-monooxygenase protein E [Bradyrhizobium sp. BTAi1]	ABQ33808	Dai	Ancylobacter polymorphus ZM13	initial hydroxylation	monooxygenase		
8	Toluene-4-monooxygenase system protein D (modular protein) [Bradyrhizobium sp. ORS 285]	SMX58036	Dai	Ancylobacter polymorphus ZM13	initial hydroxylation	monooxygenase		
9	Toluene-4-monooxygenase system protein C [Bradyrhizobium sp. ORS 285]	SMX58037	Dai	Ancylobacter polymorphus ZM13	initial hydroxylation	monooxygenase		
10	Toluene-4-monooxygenase system protein B (modular protein) (modular protein) [Bradyrhizobium sp. ORS 285]	SMX58038	Dai	Ancylobacter polymorphus ZM13	initial hydroxylation	monooxygenase		
11	Toluene-4-monooxygenase system protein A [Bradyrhizobium sp. ORS 285]	SMX58039	Dai	Ancylobacter polymorphus ZM13	initial hydroxylation	monooxygenase		
12	zinc-binding dehydrogenase [Ancylobacter sp. TS-1]	QFR34608	Dai	Ancylobacter polymorphus ZM13	intermediates metabolism	alcohol dehydrogenase		
13	Alcohol dehydrogenase GroES domain protein [Kyrpidia tusciae DSM 2912]	ADG06751	Dai	Ancylobacter polymorphus ZM13	intermediates metabolism	alcohol dehydrogenase		
14	Alcohol dehydrogenase GroES domain protein [Pectobacterium carotovorum subsp. carotovorum PC1]	ACT13397	Dai	Ancylobacter polymorphus ZM13	intermediates metabolism	alcohol dehydrogenase	K18369	adh2; alcohol dehydrogenase [EC:1.1.1.-]
15	Alcohol dehydrogenase (acceptor) [Ancylobacter novellus DSM 506]	ADH91460	Dai	Xanthobacter	intermediates metabolism	alcohol dehydrogenase		
16	Alcohol dehydrogenase zinc-binding domain protein [Xanthobacter autotrophicus Py2]	ABS67852	Dai	Xanthobacter	intermediates metabolism	alcohol dehydrogenase		
17	Alcohol dehydrogenase zinc-binding domain protein [Xanthobacter autotrophicus Py2]	ABS69096	Dai	Xanthobacter	intermediates metabolism	alcohol dehydrogenase	K00344	qor, CRYZ; NADPH:quinone reductase [EC:1.6.5.5]
18	Alcohol dehydrogenase zinc-binding domain protein [Xanthobacter autotrophicus Py2]	ABS65664	Dai	Xanthobacter	intermediates metabolism	alcohol dehydrogenase	K00344	qor, CRYZ; NADPH:quinone reductase [EC:1.6.5.5]
19	alcohol dehydrogenase [Aminobacter aminovorans]	AMS40612	Dai	Mesorhizobium	intermediates metabolism	alcohol dehydrogenase	K00121	frmA, ADH5, adhC; S-(hydroxymethyl)glutathione dehydrogenase / alcohol dehydrogenase [EC:1.1.1.284 1.1.1.1]
20	Psed_6975 | Betaine-aldehyde dehydrogenase (plasmid) [Pseudonocardia dioxanivorans CB1190]	AEA29037	Grostern	CB1190	upregulated with dioxane	aldehyde dehydrogenase	K00128	ALDH; aldehyde dehydrogenase (NAD+) [EC:1.2.1.3]
21	Psed_6981 | Aldehyde Dehydrogenase (plasmid) [Pseudonocardia dioxanivorans CB1190]	AEA29042	Grostern	CB1190	upregulated with dioxane	aldehyde dehydrogenase		
22	Aldehyde dehydrogenase family protein		Chen	multiple species	intermediates metabolism	aldehyde dehydrogenase	K00483	hpaB; 4-hydroxyphenylacetate 3-monooxygenase [EC:1.14.14.9]
23	Acetaldehyde dehydrogenase 2 [Aminobacter sp. MSH1]	AWC23184	Dai	Xanthobacter	intermediates metabolism	aldehyde dehydrogenase	K00138	aldB; aldehyde dehydrogenase [EC:1.2.1.-]
24	Psed_6971 | Hydroxyacid-oxoacid transhydrogenase (plasmid) [Pseudonocardia dioxanivorans CB1190]	AEA29033	Grostern	CB1190	upregulated with dioxane	aldehyde reductase; alcohol oxidoreductase	K11173	ADHFE1; hydroxyacid-oxoacid transhydrogenase [EC:1.1.99.24]
25	Psed_6782 | Hydroxyacid-oxoacid transhydrogenase (plasmid) [Pseudonocardia dioxanivorans CB1190]	AEA28861	Grostern	CB1190	upregulated with dioxane AND glycolate	alcohol oxidoreductase	K11173	ADHFE1; hydroxyacid-oxoacid transhydrogenase [EC:1.1.99.24]
26	N-acetylmuramic acid 6-phosphate etherase		Chen	multiple species	intermediates metabolism	etherase	K07497	K07497; putative transposase
27	Psed_4788 | D-lactate dehydrogenase (cytochrome) [Pseudonocardia dioxanivorans CB1190]	AEA26934	Grostern	CB1190	upregulated with dioxane AND glycolate	glycolate oxidase	K00104	glcD; glycolate dehydrogenase FAD-linked subunit [EC:1.1.99.14]
28	Psed_4789 | FAD linked oxidase domain protein [Pseudonocardia dioxanivorans CB1190]	AEA26935	Grostern	CB1190	upregulated with dioxane AND glycolate	glycolate oxidase	K11472	glcE; glycolate dehydrogenase FAD-binding subunit [EC:1.1.99.14]
29	Psed_4790 | protein of unknown function DUF224 cysteine-rich region domain protein [Pseudonocardia dioxanivorans CB1190]	AEA26936	Grostern	CB1190	upregulated with dioxane AND glycolate	glycolate oxidase	K11473	glcF; glycolate dehydrogenase iron-sulfur subunit [EC:1.1.99.14]
30	glycolate oxidase subunit GlcE		Chen	Xanthobacter	intermediates metabolism	glycolate oxidase	K00799	GST, gst; glutathione S-transferase [EC:2.5.1.18]
31	glycolate oxidase subunit GlcF [Microvirga thermotolerans]	QFU15682	Dai	Ancylobacter polymorphus ZM13	glycolate, glyoxylate metabolism	glycolate oxidase	K11473	glcF; glycolate dehydrogenase iron-sulfur subunit [EC:1.1.99.14]
32	FAD-binding oxidoreductase [Methylobacterium terrae]	AWN45364	Dai	Ancylobacter polymorphus ZM13	glycolate, glyoxylate metabolism	glycolate oxidase	K00104	glcD; glycolate dehydrogenase FAD-linked subunit [EC:1.1.99.14]
33	protein of unknown function DUF224 cysteine-rich region domain protein [Ancylobacter novellus DSM 506]	ADH91164	Dai	Ancylobacter polymorphus ZM13	glycolate, glyoxylate metabolism	glycolate oxidase	K11473	glcF; glycolate dehydrogenase iron-sulfur subunit [EC:1.1.99.14]
34	FAD-binding protein [Ancylobacter sp. TS-1]	QFR34008	Dai	Ancylobacter polymorphus ZM13	glycolate, glyoxylate metabolism	glycolate oxidase	K00104	glcD; glycolate dehydrogenase FAD-linked subunit [EC:1.1.99.14]
35	malate synthase G [Ancylobacter sp. TS-1]	QFR31795	Dai	Ancylobacter polymorphus ZM13	glycolate, glyoxylate metabolism	glycolate oxidase	K01638	aceB, glcB; malate synthase [EC:2.3.3.9]
36	glycolate oxidase subunit GlcD [Mesorhizobium amorphae CCNWGS0123]	ANT50121	Dai	Ancylobacter polymorphus ZM13	glycolate, glyoxylate metabolism	glycolate oxidase	K00104	glcD; glycolate dehydrogenase FAD-linked subunit [EC:1.1.99.14]
37	2-hydroxy-acid oxidase [Mesorhizobium amorphae CCNWGS0123]	ANT50122	Dai	Ancylobacter polymorphus ZM13	glycolate, glyoxylate metabolism	glycolate oxidase	K11472	glcE; glycolate dehydrogenase FAD-binding subunit [EC:1.1.99.14]
38	Lactate utilization protein A [Aminobacter sp. MSH1]	AWC25252	Dai	Ancylobacter polymorphus ZM13	glycolate, glyoxylate metabolism	glycolate oxidase	K11473	glcF; glycolate dehydrogenase iron-sulfur subunit [EC:1.1.99.14]
39	protein of unknown function DUF224 cysteine-rich region domain protein [Xanthobacter autotrophicus Py2]	ABS67113	Dai	Xanthobacter	glycolate, glyoxylate metabolism	glycolate oxidase	K11473	glcF; glycolate dehydrogenase iron-sulfur subunit [EC:1.1.99.14]
40	FAD linked oxidase domain protein [Xanthobacter autotrophicus Py2]	ABS67114	Dai	Xanthobacter	glycolate, glyoxylate metabolism	glycolate oxidase	K11472	glcE; glycolate dehydrogenase FAD-binding subunit [EC:1.1.99.14]
41	protein of unknown function DUF224 cysteine-rich region domain protein [Mesorhizobium ciceri biovar biserrulae WSM1271]	ADV11669	Dai	Mesorhizobium	glycolate, glyoxylate metabolism	glycolate oxidase	K11473	glcF; glycolate dehydrogenase iron-sulfur subunit [EC:1.1.99.14]
42	glycolate oxidase subunit GlcE [Mesorhizobium sp. M9A.F.Ca.ET.002.03.1.2]	AZO01119	Dai	Mesorhizobium	glycolate, glyoxylate metabolism	glycolate oxidase	K11472	glcE; glycolate dehydrogenase FAD-binding subunit [EC:1.1.99.14]
43	FAD-binding oxidoreductase [Mesorhizobium sp. Pch-S]	QAZ42403	Dai	Mesorhizobium	glycolate, glyoxylate metabolism	glycolate oxidase	K00104	glcD; glycolate dehydrogenase FAD-linked subunit [EC:1.1.99.14]
44	glycolate oxidase subunit GlcE (plasmid) [Sinorhizobium americanum]	APG95277	Dai	Mesorhizobium	glycolate, glyoxylate metabolism	glycolate oxidase	K11472	glcE; glycolate dehydrogenase FAD-binding subunit [EC:1.1.99.14]
45	glycolate oxidase subunit GlcD (plasmid) [Sinorhizobium americanum]	APG95278	Dai	Mesorhizobium	glycolate, glyoxylate metabolism	glycolate oxidase	K00104	glcD; glycolate dehydrogenase FAD-linked subunit [EC:1.1.99.14]
46	Psed_3890 | glyoxylate carboligase [Pseudonocardia dioxanivorans CB1190]	AEA26058	Grostern	CB1190	upregulated with dioxane AND glycolate	glyoxylate carboligase	K01608	gcl; tartronate-semialdehyde synthase [EC:4.1.1.47]
47	glyoxylate carboligase [Ancylobacter sp. TS-1]	QFR33238	Dai	Ancylobacter polymorphus ZM13	glycolate, glyoxylate metabolism	glyoxylate carboligase	K01608	gcl; tartronate-semialdehyde synthase [EC:4.1.1.47]
48	glyoxylate carboligase [Xanthobacter autotrophicus Py2]	ABS67610	Dai	Xanthobacter	glycolate, glyoxylate metabolism	glyoxylate carboligase	K01608	gcl; tartronate-semialdehyde synthase [EC:4.1.1.47]
49	Psed_3891 | glycerate kinase [Pseudonocardia dioxanivorans CB1190]	AEA26059	Grostern	CB1190	upregulated with dioxane AND glycolate	glycerate kinase	K00865	glxK, garK; glycerate 2-kinase [EC:2.7.1.165]
50	Psed_4782 | Malate synthase [Pseudonocardia dioxanivorans CB1190]	AEA26928	Grostern	CB1190	upregulated with dioxane AND glycolate	malate synthase	K01638	aceB, glcB; malate synthase [EC:2.3.3.9]
51	malate synthase G [Mesorhizobium sp. Pch-S]	QAZ41945	Dai	Ancylobacter polymorphus ZM13	glycolate, glyoxylate metabolism	malate synthase	K01638	aceB, glcB; malate synthase [EC:2.3.3.9]
52	malate synthase A [Azorhizobium caulinodans ORS 571]	BAF86076	Dai	Xanthobacter	glycolate, glyoxylate metabolism	malate synthase	K01638	aceB, glcB; malate synthase [EC:2.3.3.9]
53	malate synthase G [Xanthobacter autotrophicus Py2]	ABS69473	Dai	Mesorhizobium	glycolate, glyoxylate metabolism	malate synthase	K01638	aceB, glcB; malate synthase [EC:2.3.3.9]
54	2-isopropylmalate synthase [Ancylobacter sp. TS-1]	QFR32767	Dai	Ancylobacter polymorphus ZM13	glycolate, glyoxylate metabolism	2-isopropylmalate synthase	K01649	leuA, IMS; 2-isopropylmalate synthase [EC:2.3.3.13]
55	2-isopropylmalate synthase/homocitrate synthase family protein [Xanthobacter autotrophicus Py2]	ABS69934	Dai	Xanthobacter	glycolate, glyoxylate metabolism	2-isopropylmalate synthase	K01649	leuA, IMS; 2-isopropylmalate synthase [EC:2.3.3.13]
56	2-isopropylmalate synthase/homocitrate synthase family protein [Mesorhizobium opportunistum WSM2075]	AEH89703	Dai	Mesorhizobium	glycolate, glyoxylate metabolism	2-isopropylmalate synthase	K01649	leuA, IMS; 2-isopropylmalate synthase [EC:2.3.3.13]

S1			S2			S3			S4			S5			S6			
# hits	min % ID	max % ID	# hits	min % ID	max % ID	# hits	min % ID	max % ID	# hits	min % ID	max % ID	# hits	min % ID	max % ID	# hits	min % ID	max % ID	
30	32.418	98.619	23	34.463	98.619	25	32.418	98.619	33	33.14	98.619	29	34.561	98.619	27	32.849	98.619	1
9	32.945	98.844	7	33.841	100	8	32.945	99.065	7	32.945	98.844	10	32.209	98.844	9	32.945	98.844	2
4	76.923	98.291	4	76.923	98.291	4	76.923	98.291	4	73.729	98.291	3	76.923	98.291	4	75.424	98.291	3
30	39.627	67.573	28	40.246	74.403	32	39.394	72.656	30	40.994	67.573	33	40.994	68.627	32	40.994	73.723	4
0	N/A	N/A	0	N/A	N/A	0	N/A	N/A	0	N/A	N/A	0	N/A	N/A	0	N/A	N/A	5
5	34.412	49.851	5	36.522	49.552	4	35.652	49.552	5	36.499	49.851	6	36.499	49.552	4	36.499	49.552	6
11	35.463	49.083	9	35.577	49.213	10	35.463	47.006	13	35.463	47.006	13	35.463	45.066	14	35.463	47.006	7
0	N/A	N/A	0	N/A	N/A	0	N/A	N/A	0	N/A	N/A	0	N/A	N/A	0	N/A	N/A	8
0	N/A	N/A	0	N/A	N/A	0	N/A	N/A	0	N/A	N/A	0	N/A	N/A	0	N/A	N/A	9
0	N/A	N/A	0	N/A	N/A	0	N/A	N/A	0	N/A	N/A	0	N/A	N/A	0	N/A	N/A	10
29	38.934	66.463	27	38.923	75.427	33	39.468	74.219	30	40.486	66.667	33	40.486	68.473	31	40.491	72.993	11
101	30.633	88.542	104	30.633	88.298	108	32.115	87.069	119	31.298	85.714	125	31.298	86.42	107	31.298	90	12
47	31.043	63.277	49	31.043	63.277	50	30.89	64.241	49	31.043	63.277	51	31.043	63.277	46	31.043	63.277	13
81	31.139	61.303	79	31.139	62.651	80	31.378	59.211	93	30.239	60.541	99	29.744	61.111	90	31.339	64.615	14
0	N/A	N/A	0	N/A	N/A	0	N/A	N/A	0	N/A	N/A	0	N/A	N/A	0	N/A	N/A	15
315	32.552	63.764	361	32.353	63.91	332	32.552	63.758	376	31.755	65.015	384	32.552	64.526	357	32.19	63.2	16
208	35.938	70.588	232	35.938	69.718	225	35.938	70.303	255	35.938	70.073	283	35.938	86.802	255	35.938	89.313	17
181	35.385	65.649	213	35.224	59.752	199	35.241	65.152	219	34.769	60.697	232	35.224	59.752	210	35.241	61.943	18
366	30.982	91.892	426	30.982	94.737	396	30.982	92.444	422	30.982	92.558	488	30.982	95.376	412	30.982	90.753	19
500	36.095	100	500	36.238	99.6	500	36.095	99.6	500	36.714	99.6	500	36.095	99.6	500	36.714	99.6	20
461	29.132	98.864	500	29.132	100	489	29.132	99.643	500	29.132	98.33	500	29.362	98.33	500	29.132	98.33	21
36	35.65	75.701	39	42.795	71.292	37	43.84	73.077	37	34.018	72.881	48	34.018	73.939	39	34.018	73.77	22
500	32.438	91.718	500	34.151	94.915	500	33.264	89.905	500	33.058	88.285	500	34.519	88.106	500	34.077	89.726	23
35	41.784	99.123	33	40.515	98.834	39	40.144	100	39	39.904	98.834	38	39.068	98.834	35	41.784	98.889	24
35	33.252	67.939	32	33.252	67.381	41	33.252	75.397	41	34.709	75.694	44	33.496	67.381	35	34.709	68.182	25
1	46.065	46.065	1	46.065	46.065	1	46.065	46.065	2	46.065	48.193	1	46.065	46.065	2	46.065	53.304	26
318	29.762	85.57	369	29.762	84.058	330	29.524	84.058	378	30.087	84.273	392	29.762	84.762	357	30.238	85.612	27
11	41.624	73.575	8	41.624	73.575	12	41.624	77.297	11	41.624	73.575	12	41.624	77.033	11	41.624	73.575	28
129	33.806	87.059	171	33.952	90.351	154	33.806	86.916	179	33.645	89	197	33.806	86.946	164	33.806	87.926	29
48	43.668	64.783	62	43.668	65.368	47	44.444	65.198	53	43.913	65.368	59	43.913	65.368	59	43.913	65.368	30
183	31.354	81.081	220	31.591	78.448	215	31.354	79.798	224	31.354	80.189	259	31.354	78.378	220	31.591	81.731	31
296	27.416	76.667	335	27.416	80.153	302	27.416	72.17	348	28.169	75.229	392	27.983	71.756	339	28.095	77.273	32
178	33.654	82.883	216	32.692	81.897	208	33.654	77.778	219	33.654	79.245	249	33.654	81.197	216	33.654	80.769	33
351	29.122	83.81	386	29.122	84.404	346	29.122	87.129	420	29.122	83.036	451	29.122	85.47	384	29.691	84.545	34
149	48.638	78.226	173	48.511	81.373	144	47.737	76.068	149	47.66	76	194	50	77.005	173	49.129	76.226	35
339	28.298	92.164	366	28.298	95.652	330	28.298	90.964	393	28.105	91.603	424	28.105	97.143	371	27.79	93.631	36
77	36.829	81.212	91	36.567	85.897	80	36.829	88.679	110	36.364	90.395	111	36.145	84.173	97	36.609	90.654	37
178	33.684	85.119	217	31.798	88.35	207	31.567	83.974	221	33.732	85.965	252	33.732	90.598	217	33.492	89.216	38
169	32.203	86.598	207	31.735	80.172	199	32.203	78.261	214	32.203	77.439	235	32.203	86.325	209	32.203	87.903	39
71	35.678	68.382	85	34.739	70.701	73	35.678	70.339	100	35.678	69.565	97	35.539	68.293	90	35.678	62.376	40
178	33.017	83.817	219	32.118	86.408	210	32.143	85.217	219	33.017	85.567	253	33.017	88.889	216	33.017	88.235	41
76	35.589	81.212	92	34.826	87.179	81	35.427	86.792	110	35.589	91.241	110	35.589	82.014	95	35.411	91.589	42
347	29.048	89.683	376	28.228	92.754	337	29.048	90.833	404	28.664	92.453	435	28.664	96.19	378	29.149	91.72	43
47	34.84	65.972	65	34.748	67.391	48	34.84	63.303	73	34.84	75.63	71	34.84	75	77	34.84	62.06	44
313	28.571	81.938	355	28.571	85.294	329	28.571	87.023	370	28.571	81.95	401	28.571	81.95	356	28.571	82.609	45
252	25.874	88.889	294	25.874	90.72	265	25.874	88.777	289	25.874	90.72	293	25.874	92.5	280	25.874	90.53	46
260	26.117	88.8	311	26.117	88.235	272	26.117	93.694	296	26.117	91.339	295	26.117	85.401	284	26.117	94.915	47
290	25.812	89.516	327	25.812	89.167	292	25.812	94.595	332	25.812	91.339	332	25.812	88.321	318	25.812	92.857	48
31	33.973	82.713	36	33.973	84.043	39	33.973	82.447	38	36.963	82.447	38	33.973	82.447	40	37.088	82.447	49
119	45.247	81.532	144	45.29	89.764	120	46.301	89.908	121	43.077	84.615	159	43.191	89.764	139	44.203	81.761	50
151	45.736	82.878	175	47.581	86.538	152	46.332	89.583	154	45.614	88.664	188	45.283	90.972	176	47.778	87.603	51
118	30.357	73.711	124	29.875	79.221	121	30.357	74.104	147	30.357	74.885	183	30.357	78.632	125	30.241	77.57	52
153	50	82.857	180	50	86.076	154	51.293	88.732	161	49.573	87.156	193	51.282	86.806	187	52.778	87.742	53
227	31.323	83.673	268	30.741	88.785	239	30.556	85.849	281	33.766	80.315	334	33.499	79.265	287	31.323	83.168	54
157	40.72	80.645	192	39.943	78.261	160	31.296	80	209	39.514	78.846	239	31.296	79.459	205	40.439	82.308	55
152	40.816	87.683	196	40.154	89.381	157	39.931	88.265	215	39.744	88.406	234	29.735	90.833	212	41.111	93.069	56

Table S5. Summary of BLASTp hits results for 1,4-dioxane degradation pathway enzymes in Lowry support media (2-4). Red rows indicate queries that had no hits in the Lowry samples. Blue rows and blue values indicate amino acid identities of 90+%.

									S7			S8			S9			S10			S11			S12			
	Query (1,4-dioxane degradation pathway enzyme proteins)	Accession	Paper	Strain	Function	Pathway Enzyme	KO	Definition	# hits	min % ID	max % ID	# hits	min % ID	max % ID	# hits	min % ID	max % ID	# hits	min % ID	max % ID	# hits	min % ID	max % ID	# hits	min % ID	max % ID	
1	Psed_6977 | Ferredoxin--NAD(+) reductase (plasmid) [Pseudonocardia dioxanivorans CB1190]	AEA29038	Grostern	CB1190	upregulated with dioxane	monooxygenase	K18225	prmB; propane monooxygenase reductase component [EC:1.18.1.-]	25	34.561	98.619	24	33.14	98.619	27	33.14	98.619	25	34.426	98.619	29	34.463	98.619	32	34.463	99.306	1
2	Psed_6978 | methane/phenol/toluene hydroxylase (plasmid) [Pseudonocardia dioxanivorans CB1190]	AEA29039	Grostern	CB1190	upregulated with dioxane	monooxygenase	K18224	prmC; propane 2-monooxygenase small subunit [EC:1.14.13.227]	9	32.209	99.057	7	32.803	98.844	7	32.945	98.844	9	32.209	98.844	9	32.209	98.844	10	32.209	98.844	2
3	Psed_6979 | monooxygenase component MmoB/DmpM (plasmid) [Pseudonocardia dioxanivorans CB1190]	AEA29040	Grostern	CB1190	upregulated with dioxane	monooxygenase			3	76.923	98.291	4	75.424	98.291	4	75.424	98.291	4	75.424	98.291	3	76.923	98.291	4	75.424	98.291	3
4	YHS protein		Chen	Rhizobiales	initial hydroxylation	monooxygenase	K15760	tmoA, tbuA1, touA; toluene monooxygenase system protein A [EC:1.14.13.236 1.14.13.-]	34	40.994	73.864	32	40.47	67.949	31	40.994	68.153	32	40.994	71.429	36	40.441	71.2	35	39.348	67.573	4
5	4-Hydroxyphenylacetate 3-hydroxylase		Chen	Xanthobacter	initial hydroxylation	monooxygenase			0	N/A	N/A	0	N/A	N/A	0	N/A	N/A	0	N/A	N/A	0	N/A	N/A	0	N/A	N/A	5
6	putative ferredoxin NAD(+) reductase [Bradyrhizobium sp. BTAi1]	ABQ33807	Dai	Ancylobacter polymorphus ZM13	initial hydroxylation	monooxygenase			4	36.499	49.552	5	36.499	49.552	5	36.499	49.552	6	36.283	49.552	7	36.283	49.552	7	36.283	49.552	6
7	toluene 4-monooxygenase protein E [Bradyrhizobium sp. BTAi1]	ABQ33808	Dai	Ancylobacter polymorphus ZM13	initial hydroxylation	monooxygenase			15	35.463	49.51	13	35.463	47.006	14	35.463	47.006	15	35.463	47.006	18	35.463	47.006	16	35.463	47.006	7
8	Toluene-4-monooxygenase system protein D (modular protein) [Bradyrhizobium sp. ORS 285]	SMX58036	Dai	Ancylobacter polymorphus ZM13	initial hydroxylation	monooxygenase			0	N/A	N/A	0	N/A	N/A	0	N/A	N/A	0	N/A	N/A	0	N/A	N/A	0	N/A	N/A	8
9	Toluene-4-monooxygenase system protein C [Bradyrhizobium sp. ORS 285]	SMX58037	Dai	Ancylobacter polymorphus ZM13	initial hydroxylation	monooxygenase			0	N/A	N/A	0	N/A	N/A	0	N/A	N/A	0	N/A	N/A	0	N/A	N/A	0	N/A	N/A	9
10	Toluene-4-monooxygenase system protein B (modular protein) (modular protein) [Bradyrhizobium sp. ORS 285]	SMX58038	Dai	Ancylobacter polymorphus ZM13	initial hydroxylation	monooxygenase			0	N/A	N/A	0	N/A	N/A	0	N/A	N/A	0	N/A	N/A	0	N/A	N/A	0	N/A	N/A	10
11	Toluene-4-monooxygenase system protein A [Bradyrhizobium sp. ORS 285]	SMX58039	Dai	Ancylobacter polymorphus ZM13	initial hydroxylation	monooxygenase			34	40.491	76.136	30	40.491	69.006	31	40.486	69.364	32	40.486	73.451	37	40.441	72.83	35	40.491	66.667	11
12	zinc-binding dehydrogenase [Ancylobacter sp. TS-1]	QFR34608	Dai	Ancylobacter polymorphus ZM13	intermediates metabolism	alcohol dehydrogenase			113	31.298	87.129	119	31.298	87.121	134	31.298	86.869	132	31.298	87.755	139	31.298	86.667	119	31.298	83.898	12
13	Alcohol dehydrogenase GroES domain protein [Kyrpidia tusciae DSM 2912]	ADG06751	Dai	Ancylobacter polymorphus ZM13	intermediates metabolism	alcohol dehydrogenase			46	31.043	63.277	49	31.043	63.277	59	31.043	63.277	50	31.043	63.277	50	31.043	63.277	50	31.043	63.277	13
14	Alcohol dehydrogenase GroES domain protein [Pectobacterium carotovorum subsp. carotovorum PC1]	ACT13397	Dai	Ancylobacter polymorphus ZM13	intermediates metabolism	alcohol dehydrogenase	K18369	adh2; alcohol dehydrogenase [EC:1.1.1.-]	96	31.339	64.103	96	31.282	63.793	98	31.282	59.627	90	30.352	59.22	104	29.744	59.901	88	31.458	62.085	14
15	Alcohol dehydrogenase (acceptor) [Ancylobacter novellus DSM 506]	ADH91460	Dai	Xanthobacter	intermediates metabolism	alcohol dehydrogenase			0	N/A	N/A	0	N/A	N/A	0	N/A	N/A	0	N/A	N/A	0	N/A	N/A	0	N/A	N/A	15
16	Alcohol dehydrogenase zinc-binding domain protein [Xanthobacter autotrophicus Py2]	ABS67852	Dai	Xanthobacter	intermediates metabolism	alcohol dehydrogenase			377	32.552	63.514	359	32.552	65.414	439	31.034	63.467	411	32.552	65.333	473	32.552	63.226	419	32.454	64.394	16
17	Alcohol dehydrogenase zinc-binding domain protein [Xanthobacter autotrophicus Py2]	ABS69096	Dai	Xanthobacter	intermediates metabolism	alcohol dehydrogenase	K00344	qor, CRYZ; NADPH:quinone reductase [EC:1.6.5.5]	272	35.938	70.968	264	35.938	68.992	309	35.938	87.218	293	35.938	69.748	323	35.938	69.412	291	35.938	75.61	17
18	Alcohol dehydrogenase zinc-binding domain protein [Xanthobacter autotrophicus Py2]	ABS65664	Dai	Xanthobacter	intermediates metabolism	alcohol dehydrogenase	K00344	qor, CRYZ; NADPH:quinone reductase [EC:1.6.5.5]	225	35.224	62.428	214	35.241	63.102	242	35.224	59.752	225	35.241	62.821	283	34.328	68.063	259	35.224	62.032	18
19	alcohol dehydrogenase [Aminobacter aminovorans]	AMS40612	Dai	Mesorhizobium	intermediates metabolism	alcohol dehydrogenase	K00121	frmA, ADH5, adhC; S-(hydroxymethyl)glutathione dehydrogenase / alcohol dehydrogenase [EC:1.1.1.284 1.1.1.1]	440	30.982	93.793	435	30.982	91.667	500	30.982	93.269	481	30.982	91.209	500	31.452	94.681	500	30.982	93.443	19
20	Psed_6975 | Betaine-aldehyde dehydrogenase (plasmid) [Pseudonocardia dioxanivorans CB1190]	AEA29037	Grostern	CB1190	upregulated with dioxane	aldehyde dehydrogenase	K00128	ALDH; aldehyde dehydrogenase (NAD+) [EC:1.2.1.3]	500	36.714	99.6	500	36.714	99.6	500	36.714	99.6	500	36.095	99.627	500	36.714	99.6	500	36.714	99.6	20
21	Psed_6981 | Aldehyde Dehydrogenase (plasmid) [Pseudonocardia dioxanivorans CB1190]	AEA29042	Grostern	CB1190	upregulated with dioxane	aldehyde dehydrogenase			500	29.449	98.33	500	28.964	98.33	500	29.959	100	500	29.661	98.33	500	29.959	98.473	500	29.832	98.33	21
22	Aldehyde dehydrogenase family protein		Chen	multiple speces	intermediates metabolism	aldehyde dehydrogenase	K00483	hpaB; 4-hydroxyphenylacetate 3-monooxygenase [EC:1.14.14.9]	44	34.018	73.394	37	35.952	73.171	42	34.018	71.901	41	34.018	70.044	50	36.254	72.832	45	34.018	73.103	22
23	Acetaldehyde dehydrogenase 2 [Aminobacter sp. MSH1]	AWC23184	Dai	Xanthobacter	intermediates metabolism	aldehyde dehydrogenase	K00138	aldB; aldehyde dehydrogenase [EC:1.2.1.-]	500	34.077	94.969	500	33.74	89.697	500	34.623	91.477	500	34.151	91.772	500	34.151	91.089	500	34.623	92.585	23
24	Psed_6971 | Hydroxyacid-oxoacid transhydrogenase (plasmid) [Pseudonocardia dioxanivorans CB1190]	AEA29033	Grostern	CB1190	upregulated with dioxane	aldehyde reductase; alcohol oxidoreductase	K11173	ADHFE1; hydroxyacid-oxoacid transhydrogenase [EC:1.1.99.24]	42	33.588	98.834	35	40.385	98.834	42	40.521	99.342	44	32.85	98.834	41	38.686	98.834	40	41.784	98.917	24
25	Psed_6782 | Hydroxyacid-oxoacid transhydrogenase (plasmid) [Pseudonocardia dioxanivorans CB1190]	AEA28861	Grostern	CB1190	upregulated with dioxane AND glycolate	alcohol oxidoreductase	K11173	ADHFE1; hydroxyacid-oxoacid transhydrogenase [EC:1.1.99.24]	43	33.168	68.667	36	34.709	68.519	42	34.709	67.381	47	33.252	68.595	47	33.168	67.381	44	34.709	76.033	25
26	N-acetylmuramic acid 6-phosphate etherase		Chen	multiple speces	intermediates metabolism	etherase	K07497	K07497; putative transposase	1	46.065	46.065	1	46.065	46.065	1	46.224	46.224	2	46.065	54.819	1	46.065	46.065	3	46.065	54.335	26
27	Psed_4788 | D-lactate dehydrogenase (cytochrome) [Pseudonocardia dioxanivorans CB1190]	AEA26934	Grostern	CB1190	upregulated with dioxane AND glycolate	glycolate oxidase	K00104	glcD; glycolate dehydrogenase FAD-linked subunit [EC:1.1.99.14]	371	29.524	84.058	377	30.238	84.058	432	29.762	84.265	403	29.762	84.265	446	29.762	87.166	434	29.594	84.821	27
28	Psed_4789 | FAD linked oxidase domain protein [Pseudonocardia dioxanivorans CB1190]	AEA26935	Grostern	CB1190	upregulated with dioxane AND glycolate	glycolate oxidase	K11472	glcE; glycolate dehydrogenase FAD-binding subunit [EC:1.1.99.14]	11	41.624	73.575	10	41.624	73.575	11	41.624	73.575	12	41.624	73.575	14	41.624	73.575	13	41.624	73.575	28
29	Psed_4790 | protein of unknown function DUF224 cysteine-rich region domain protein [Pseudonocardia dioxanivorans CB1190]	AEA26936	Grostern	CB1190	upregulated with dioxane AND glycolate	glycolate oxidase	K11473	glcF; glycolate dehydrogenase iron-sulfur subunit [EC:1.1.99.14]	176	33.645	86.881	183	33.645	85.979	205	32.918	86.946	182	33.645	87.413	231	33.645	91.228	207	33.645	86.946	29
30	glycolate oxidase subunit GlcE		Chen	Xanthobacter	intermediates metabolism	glycolate oxidase	K00799	GST, gst; glutathione S-transferase [EC:2.5.1.18]	59	44.269	65.368	60	43.913	66.372	66	43.913	65.368	57	43.913	65.368	62	43.913	65.652	65	42.544	65.854	30
31	glycolate oxidase subunit GlcF [Microvirga thermotolerans]	QFU15682	Dai	Ancylobacter polymorphus ZM13	glycolate, glyoxylate metabolism	glycolate oxidase	K11473	glcF; glycolate dehydrogenase iron-sulfur subunit [EC:1.1.99.14]	235	31.591	83.333	225	31.591	77.982	269	31.591	77.982	251	31.591	85.149	289	31.591	84.536	265	31.368	83.168	31
32	FAD-binding oxidoreductase [Methylobacterium terrae]	AWN45364	Dai	Ancylobacter polymorphus ZM13	glycolate, glyoxylate metabolism	glycolate oxidase	K00104	glcD; glycolate dehydrogenase FAD-linked subunit [EC:1.1.99.14]	347	28.095	75	354	27.983	81.295	393	27.983	73.611	384	27.983	73.78	411	27.983	74.138	417	27.983	71.472	32
33	protein of unknown function DUF224 cysteine-rich region domain protein [Ancylobacter novellus DSM 506]	ADH91164	Dai	Ancylobacter polymorphus ZM13	glycolate, glyoxylate metabolism	glycolate oxidase	K11473	glcF; glycolate dehydrogenase iron-sulfur subunit [EC:1.1.99.14]	229	33.254	83.784	227	33.254	79.07	267	33.654	81.25	243	33.254	84.848	279	32.952	85.567	252	32.692	84	33
34	FAD-binding protein [Ancylobacter sp. TS-1]	QFR34008	Dai	Ancylobacter polymorphus ZM13	glycolate, glyoxylate metabolism	glycolate oxidase	K00104	glcD; glycolate dehydrogenase FAD-linked subunit [EC:1.1.99.14]	397	29.122	87.129	401	29.122	86.325	456	28.791	86.29	446	29.122	83.099	487	29.122	83.495	473	29.122	86.667	34
35	malate synthase G [Ancylobacter sp. TS-1]	QFR31795	Dai	Ancylobacter polymorphus ZM13	glycolate, glyoxylate metabolism	glycolate oxidase	K01638	aceB, glcB; malate synthase [EC:2.3.3.9]	159	49.874	76.168	174	50.216	79.13	177	50.233	78.505	161	49.749	77.444	188	48.485	75.439	167	48.207	75	35
36	glycolate oxidase subunit GlcD [Mesorhizobium amorphae CCNWGS0123]	ANT50121	Dai	Ancylobacter polymorphus ZM13	glycolate, glyoxylate metabolism	glycolate oxidase	K00104	glcD; glycolate dehydrogenase FAD-linked subunit [EC:1.1.99.14]	381	28.105	93.373	386	28.105	97.122	429	27.79	98.182	422	28.105	96.599	460	28.105	94.057	458	28.105	94.444	36
37	2-hydroxy-acid oxidase [Mesorhizobium amorphae CCNWGS0123]	ANT50122	Dai	Ancylobacter polymorphus ZM13	glycolate, glyoxylate metabolism	glycolate oxidase	K11472	glcE; glycolate dehydrogenase FAD-binding subunit [EC:1.1.99.14]	99	36.829	85.821	91	36.145	85.811	118	37.073	87.562	102	36.145	78.947	118	36.145	78.832	101	36.145	84.167	37
38	Lactate utilization protein A [Aminobacter sp. MSH1]	AWC25252	Dai	Ancylobacter polymorphus ZM13	glycolate, glyoxylate metabolism	glycolate oxidase	K11473	glcF; glycolate dehydrogenase iron-sulfur subunit [EC:1.1.99.14]	225	33.732	85.185	228	33.732	84.892	266	33.732	92.708	243	33.732	89.209	279	32.867	91.743	255	33.732	91	38
39	protein of unknown function DUF224 cysteine-rich region domain protein [Xanthobacter autotrophicus Py2]	ABS67113	Dai	Xanthobacter	glycolate, glyoxylate metabolism	glycolate oxidase	K11473	glcF; glycolate dehydrogenase iron-sulfur subunit [EC:1.1.99.14]	215	32.203	87.273	218	32.203	83.486	260	32.203	83.333	237	32.203	85.859	271	32.203	87.629	243	31.735	86	39
40	FAD linked oxidase domain protein [Xanthobacter autotrophicus Py2]	ABS67114	Dai	Xanthobacter	glycolate, glyoxylate metabolism	glycolate oxidase	K11472	glcE; glycolate dehydrogenase FAD-binding subunit [EC:1.1.99.14]	95	35.678	67.284	81	35.539	68.824	105	35.678	69.173	93	35.539	70.13	109	35.539	70.064	97	35.539	68.345	40
41	protein of unknown function DUF224 cysteine-rich region domain protein [Mesorhizobium ciceri biovar biserrulae WSM1271]	ADV11669	Dai	Mesorhizobium	glycolate, glyoxylate metabolism	glycolate oxidase	K11473	glcF; glycolate dehydrogenase iron-sulfur subunit [EC:1.1.99.14]	227	32.405	85.802	226	33.254	84.404	269	33.017	93.75	243	32.405	91.367	281	32.405	86.239	256	32.118	86	41
42	glycolate oxidase subunit GlcE [Mesorhizobium sp. M9A.F.Ca.ET.002.03.1.2]	AZO01119	Dai	Mesorhizobium	glycolate, glyoxylate metabolism	glycolate oxidase	K11472	glcE; glycolate dehydrogenase FAD-binding subunit [EC:1.1.99.14]	101	35.589	81.818	88	35.589	86.577	117	35.589	87	103	35.589	78.621	122	35.589	80.597	101	35.589	86.667	42
43	FAD-binding oxidoreductase [Mesorhizobium sp. Pch-S]	QAZ42403	Dai	Mesorhizobium	glycolate, glyoxylate metabolism	glycolate oxidase	K00104	glcD; glycolate dehydrogenase FAD-linked subunit [EC:1.1.99.14]	389	28.664	91.566	395	28.664	95.96	440	29.048	94.545	438	28.664	94.702	479	28.664	91.99	467	28.664	94.444	43
44	glycolate oxidase subunit GlcE (plasmid) [Sinorhizobium americanum]	APG95277	Dai	Mesorhizobium	glycolate, glyoxylate metabolism	glycolate oxidase	K11472	glcE; glycolate dehydrogenase FAD-binding subunit [EC:1.1.99.14]	69	34.84	62.06	67	34.84	62.06	78	34.84	64.751	74	34.84	69.63	85	34.84	62.06	70	33.92	68.824	44
45	glycolate oxidase subunit GlcD (plasmid) [Sinorhizobium americanum]	APG95278	Dai	Mesorhizobium	glycolate, glyoxylate metabolism	glycolate oxidase	K00104	glcD; glycolate dehydrogenase FAD-linked subunit [EC:1.1.99.14]	360	28.571	84.615	380	28.571	81.95	420	28.571	83.333	404	28.448	81.95	433	28.571	89.908	431	28.571	86.034	45
46	Psed_3890 | glyoxylate carboligase [Pseudonocardia dioxanivorans CB1190]	AEA26058	Grostern	CB1190	upregulated with dioxane AND glycolate	glyoxylate carboligase	K01608	gcl; tartronate-semialdehyde synthase [EC:4.1.1.47]	292	25.874	88.777	289	25.874	90.939	340	25.874	92.5	321	25.874	88.61	352	28.423	90.939	319	25.874	90.939	46
47	glyoxylate carboligase [Ancylobacter sp. TS-1]	QFR33238	Dai	Ancylobacter polymorphus ZM13	glycolate, glyoxylate metabolism	glyoxylate carboligase	K01608	gcl; tartronate-semialdehyde synthase [EC:4.1.1.47]	294	26.117	94.286	295	26.117	91.111	345	26.117	92.188	321	26.117	92.453	364	26.117	92.5	325	26.117	86.957	47
48	glyoxylate carboligase [Xanthobacter autotrophicus Py2]	ABS67610	Dai	Xanthobacter	glycolate, glyoxylate metabolism	glyoxylate carboligase	K01608	gcl; tartronate-semialdehyde synthase [EC:4.1.1.47]	328	25.812	95.238	323	25.812	92.437	380	25.812	91.406	354	25.812	94.34	392	25.812	96.226	362	25.812	90.517	48
49	Psed_3891 | glycerate kinase [Pseudonocardia dioxanivorans CB1190]	AEA26059	Grostern	CB1190	upregulated with dioxane AND glycolate	glycerate kinase	K00865	glxK, garK; glycerate 2-kinase [EC:2.7.1.165]	39	37.802	84.302	36	37.802	82.278	42	35.23	82.181	40	37.466	82.447	44	37.802	81.915	35	37.802	82.447	49
50	Psed_4782 | Malate synthase [Pseudonocardia dioxanivorans CB1190]	AEA26928	Grostern	CB1190	upregulated with dioxane AND glycolate	malate synthase	K01638	aceB, glcB; malate synthase [EC:2.3.3.9]	130	45.725	87.943	142	47.396	89.764	154	44.745	88.462	139	44.051	90.26	147	46.866	86.147	137	45.725	89.933	50
51	malate synthase G [Mesorhizobium sp. Pch-S]	QAZ41945	Dai	Ancylobacter polymorphus ZM13	glycolate, glyoxylate metabolism	malate synthase	K01638	aceB, glcB; malate synthase [EC:2.3.3.9]	164	47.807	87.121	179	47.284	81.347	184	47.005	94.175	174	46.024	91.667	195	48.8	92.105	171	45	87.611	51
52	malate synthase A [Azorhizobium caulinodans ORS 571]	BAF86076	Dai	Xanthobacter	glycolate, glyoxylate metabolism	malate synthase	K01638	aceB, glcB; malate synthase [EC:2.3.3.9]	149	30.357	77.619	155	30.357	74.074	149	30.357	73.333	158	30.357	79.63	184	30.357	73.78	170	30.357	73.154	52
53	malate synthase G [Xanthobacter autotrophicus Py2]	ABS69473	Dai	Mesorhizobium	glycolate, glyoxylate metabolism	malate synthase	K01638	aceB, glcB; malate synthase [EC:2.3.3.9]	174	51.2	86.364	186	51.163	84.314	189	53.503	90.291	177	50.267	86.916	206	50.5	91.15	177	50.216	84.615	53
54	2-isopropylmalate synthase [Ancylobacter sp. TS-1]	QFR32767	Dai	Ancylobacter polymorphus ZM13	glycolate, glyoxylate metabolism	2-isopropylmalate synthase	K01649	leuA, IMS; 2-isopropylmalate synthase [EC:2.3.3.13]	308	33.766	81.301	289	33.766	81.481	328	33.766	83.708	312	33.766	81.343	338	33.766	83.178	330	33.766	82.105	54
55	2-isopropylmalate synthase/homocitrate synthase family protein [Xanthobacter autotrophicus Py2]	ABS69934	Dai	Xanthobacter	glycolate, glyoxylate metabolism	2-isopropylmalate synthase	K01649	leuA, IMS; 2-isopropylmalate synthase [EC:2.3.3.13]	220	40.53	84.071	207	31.296	79.817	235	40.084	83.962	235	31.296	83.133	237	39.286	86.916	241	40.453	79.817	55
56	2-isopropylmalate synthase/homocitrate synthase family protein [Mesorhizobium opportunistum WSM2075]	AEH89703	Dai	Mesorhizobium	glycolate, glyoxylate metabolism	2-isopropylmalate synthase	K01649	leuA, IMS; 2-isopropylmalate synthase [EC:2.3.3.13]	219	39.091	92.929	204	37.838	92.157	236	39.596	91.912	228	40.063	90.426	237	40.643	87.195	242	40.947	88.776	56

Table S5. Summary of BLASTp hits results for 1,4-dioxane degradation pathway enzymes in Lowry support media (continued) (2-4). Red rows indicate queries that had no hits in the Lowry samples. Blue rows and blue values indicate amino acid identities of 90+%.

	Query (sequences upregulated in CB1190 during dioxane and glycolate degradation)	Accession	Paper	Strain	Function	KO	Definition
1	Psed_0038 | regulatory protein ArsR [Pseudonocardia dioxanivorans CB1190]	AEA22320	Grostern	CB1190	upregulated with dioxane		
2	Psed_1076 | short-chain dehydrogenase/reductase SDR [Pseudonocardia dioxanivorans CB1190]	AEA23327	Grostern	CB1190	upregulated with dioxane		
3	Psed_1594 | protein of unknown function UPF0016 [Pseudonocardia dioxanivorans CB1190]	AEA23831	Grostern	CB1190	upregulated with dioxane	K23541	TMEM165, GDT1; Ca2+/H+ antiporter, TMEM165/GDT1 family
4	Psed_1658 | Trimethylamine-N-oxide reductase (cytochrome c) [Pseudonocardia dioxanivorans CB1190]	AEA23894	Grostern	CB1190	upregulated with dioxane		
5	Psed_3041 | NADH dehydrogenase (ubiquinone) 24 kDa subunit [Pseudonocardia dioxanivorans CB1190]	AEA25240	Grostern	CB1190	upregulated with dioxane	K00127	fdoI, fdsG; formate dehydrogenase subunit gamma
6	Psed_3564 | putative transcriptional regulator, GntR family [Pseudonocardia dioxanivorans CB1190]	AEA25736	Grostern	CB1190	upregulated with dioxane	K05825	LYSN; 2-aminoadipate transaminase [EC:2.6.1.-]
7	Psed_3934 | major facilitator superfamily MFS_1 [Pseudonocardia dioxanivorans CB1190]	AEA26101	Grostern	CB1190	upregulated with dioxane		
8	Psed_3935 | transcriptional regulator, GntR family [Pseudonocardia dioxanivorans CB1190]	AEA26102	Grostern	CB1190	upregulated with dioxane		
9	Psed_4146 | Potassium-transporting ATPase B chain [Pseudonocardia dioxanivorans CB1190]	AEA26309	Grostern	CB1190	upregulated with dioxane	K01547	kdpB; potassium-transporting ATPase ATP-binding subunit [EC:7.2.2.6]
10	Psed_4147 | Potassium-transporting ATPase A chain [Pseudonocardia dioxanivorans CB1190]	AEA26310	Grostern	CB1190	upregulated with dioxane	K01546	kdpA; potassium-transporting ATPase potassium-binding subunit
11	Psed_4148 | K+-transporting ATPase, F subunit [Pseudonocardia dioxanivorans CB1190]	AEA26311	Grostern	CB1190	upregulated with dioxane		
12	Psed_5135 | malic protein NAD-binding protein [Pseudonocardia dioxanivorans CB1190]	AEA27272	Grostern	CB1190	upregulated with dioxane	K00027	ME2, sfcA, maeA; malate dehydrogenase (oxaloacetate-decarboxylating) [EC:1.1.1.38]
13	Psed_5524 | hydrolase [Pseudonocardia dioxanivorans CB1190]	AEA27654	Grostern	CB1190	upregulated with dioxane		
14	Psed_5938 | glycoside hydrolase family 13 domain-containing protein [Pseudonocardia dioxanivorans CB1190]	AEA28060	Grostern	CB1190	upregulated with dioxane	K00700	GBE1, glgB; 1,4-alpha-glucan branching enzyme [EC:2.4.1.18]
15	Psed_6259 | FMN-dependent oxidoreductase, nitrilotriacetate monooxygenase family [Pseudonocardia dioxanivorans CB1190]	AEA28360	Grostern	CB1190	upregulated with dioxane	K20938	ladA; long-chain alkane monooxygenase [EC:1.14.14.28]
16	Psed_6261 | ABC-type transporter, integral membrane subunit [Pseudonocardia dioxanivorans CB1190]	AEA28362	Grostern	CB1190	upregulated with dioxane	K01998	livM; branched-chain amino acid transport system permease protein
17	Psed_6262 | ABC-type transporter, integral membrane subunit [Pseudonocardia dioxanivorans CB1190]	AEA28363	Grostern	CB1190	upregulated with dioxane	K01997	livH; branched-chain amino acid transport system permease protein
18	Psed_6263 | Extracellular ligand-binding receptor [Pseudonocardia dioxanivorans CB1190]	AEA28364	Grostern	CB1190	upregulated with dioxane	K01999	livK; branched-chain amino acid transport system substrate-binding protein
19	Psed_6600 | acyl-CoA dehydrogenase domain-containing protein [Pseudonocardia dioxanivorans CB1190]	AEA28688	Grostern	CB1190	upregulated with dioxane		
20	Psed_6728 | Amidase (plasmid) [Pseudonocardia dioxanivorans CB1190]	AEA28809	Grostern	CB1190	upregulated with dioxane	K02433	gatA, QRSL1; aspartyl-tRNA(Asn)/glutamyl-tRNA(Gln) amidotransferase subunit A [EC:6.3.5.6 6.3.
21	Psed_6730 | Luciferase-like, subgroup (plasmid) [Pseudonocardia dioxanivorans CB1190]	AEA28811	Grostern	CB1190	upregulated with dioxane	K09018	rutA; pyrimidine oxygenase [EC:1.14.99.46]
22	Psed_6732 | MaoC domain protein dehydratase (plasmid) [Pseudonocardia dioxanivorans CB1190]	AEA28813	Grostern	CB1190	upregulated with dioxane	K18290	ich-Y; itaconyl-CoA hydratase [EC:4.2.1.56]
23	Psed_6735 | flavoprotein WrbA (plasmid) [Pseudonocardia dioxanivorans CB1190]	AEA28816	Grostern	CB1190	upregulated with dioxane	K03809	wrbA; NAD(P)H dehydrogenase (quinone) [EC:1.6.5.2]
24	Psed_6742 | NLP/P60 protein (plasmid) [Pseudonocardia dioxanivorans CB1190]	AEA28823	Grostern	CB1190	upregulated with dioxane		
25	Psed_6745 | ATP-binding protein (plasmid) [Pseudonocardia dioxanivorans CB1190]	AEA28826	Grostern	CB1190	upregulated with dioxane		
26	Psed_6751 | Transglycosylase-like domain protein (plasmid) [Pseudonocardia dioxanivorans CB1190]	AEA28832	Grostern	CB1190	upregulated with dioxane	K21687	rpfA; resuscitation-promoting factor RpfA
27	Psed_6799 | transposase IS4 family protein (plasmid) [Pseudonocardia dioxanivorans CB1190]	AEA28871	Grostern	CB1190	upregulated with dioxane		
28	Psed_6970 | D-lactate dehydrogenase (cytochrome) (plasmid) [Pseudonocardia dioxanivorans CB1190]	AEA29032	Grostern	CB1190	upregulated with dioxane		
29	Psed_6972 | transcriptional regulator, GntR family (plasmid) [Pseudonocardia dioxanivorans CB1190]	AEA29034	Grostern	CB1190	upregulated with dioxane		
30	Psed_6974 | Ethyl tert-butyl ether degradation EthD (plasmid) [Pseudonocardia dioxanivorans CB1190]	AEA29036	Grostern	CB1190	upregulated with dioxane		
31	Psed_6982 | Mn2+/Fe2+ transporter, NRAMP family (plasmid) [Pseudonocardia dioxanivorans CB1190]	AEA29043	Grostern	CB1190	upregulated with dioxane	K03322	mntH; manganese transport protein
32	Psed_7002 | Mn2+/Fe2+ transporter, NRAMP family (plasmid) [Pseudonocardia dioxanivorans CB1190]	AEA29059	Grostern	CB1190	upregulated with dioxane	K03322	mntH; manganese transport protein
33	Psed_0350 | Bile acid:sodium symporter [Pseudonocardia dioxanivorans CB1190]	AEA22623	Grostern	CB1190	upregulated with dioxane AND glycolate	K14347	SLC10A7, P7; solute carrier family 10 (sodium/bile acid cotransporter), member 7
34	Psed_1302 | protein of unknown function DUF156 [Pseudonocardia dioxanivorans CB1190]	AEA23545	Grostern	CB1190	upregulated with dioxane AND glycolate	K21600	csoR, ricR; CsoR family transcriptional regulator, copper-sensing transcriptional repressor
35	Psed_1303 | Heavy metal transport/detoxification protein [Pseudonocardia dioxanivorans CB1190]	AEA23546	Grostern	CB1190	upregulated with dioxane AND glycolate		
36	Psed_1304 | heavy metal translocating P-type ATPase [Pseudonocardia dioxanivorans CB1190]	AEA23547	Grostern	CB1190	upregulated with dioxane AND glycolate	K17686	copA, ctpA, ATP7; P-type Cu+ transporter [EC:7.2.2.8]
37	Psed_1584 | 6-phosphofructokinase [Pseudonocardia dioxanivorans CB1190]	AEA23821	Grostern	CB1190	upregulated with dioxane AND glycolate	K00850	pfkA, PFK; 6-phosphofructokinase 1 [EC:2.7.1.11]
38	Psed_2030 | Linalool 8-monooxygenase [Pseudonocardia dioxanivorans CB1190]	AEA24257	Grostern	CB1190	upregulated with dioxane AND glycolate		
39	Psed_2371 | ABC-type transporter, periplasmic subunit [Pseudonocardia dioxanivorans CB1190]	AEA24577	Grostern	CB1190	upregulated with dioxane AND glycolate	K02035	ABC.PE.S; peptide/nickel transport system substrate-binding protein
40	Psed_3522 | response regulator receiver protein [Pseudonocardia dioxanivorans CB1190]	AEA25695	Grostern	CB1190	upregulated with dioxane AND glycolate		
41	Psed_3888 | Hydroxypyruvate isomerase [Pseudonocardia dioxanivorans CB1190]	AEA26056	Grostern	CB1190	upregulated with dioxane AND glycolate	K01816	hyi, gip; hydroxypyruvate isomerase [EC:5.3.1.22]
42	Psed_3889 | 2-hydroxy-3-oxopropionate reductase [Pseudonocardia dioxanivorans CB1190]	AEA26057	Grostern	CB1190	upregulated with dioxane AND glycolate	K00042	garR, glxR; 2-hydroxy-3-oxopropionate reductase [EC:1.1.1.60]
43	Psed_4031 | iron-sulfur cluster binding protein [Pseudonocardia dioxanivorans CB1190]	AEA26196	Grostern	CB1190	upregulated with dioxane AND glycolate	K18929	lldF; L-lactate dehydrogenase complex protein LldF
44	Psed_4512 | ABC-type transporter, integral membrane subunit [Pseudonocardia dioxanivorans CB1190]	AEA26667	Grostern	CB1190	upregulated with dioxane AND glycolate	K15554	ssuC; sulfonate transport system permease protein
45	Psed_4513 | cobalamin (vitamin B12) biosynthesis CbiX protein [Pseudonocardia dioxanivorans CB1190]	AEA26668	Grostern	CB1190	upregulated with dioxane AND glycolate		
46	Psed_4577 | Cold-shock protein DNA-binding protein [Pseudonocardia dioxanivorans CB1190]	AEA26729	Grostern	CB1190	upregulated with dioxane AND glycolate		
47	Psed_4755 | ammonium transporter [Pseudonocardia dioxanivorans CB1190]	AEA26902	Grostern	CB1190	upregulated with dioxane AND glycolate	K03320	amt, AMT, MEP; ammonium transporter, Amt family
48	Psed_5025 | 4-hydroxyacetophenone monooxygenase [Pseudonocardia dioxanivorans CB1190]	AEA27163	Grostern	CB1190	upregulated with dioxane AND glycolate		
49	Psed_5026 | regulatory protein TetR [Pseudonocardia dioxanivorans CB1190]	AEA27164	Grostern	CB1190	upregulated with dioxane AND glycolate		
50	Psed_5406 | NAD(P)(+) transhydrogenase (AB-specific) [Pseudonocardia dioxanivorans CB1190]	AEA27539	Grostern	CB1190	upregulated with dioxane AND glycolate	K00324	pntA; proton-translocating NAD(P)+ transhydrogenase subunit alpha [EC:7.1.1.1]
51	Psed_5736 | Transglycosylase-like domain protein [Pseudonocardia dioxanivorans CB1190]	AEA27863	Grostern	CB1190	upregulated with dioxane AND glycolate	K21687	rpfA; resuscitation-promoting factor RpfA
52	Psed_6175 | regulatory protein TetR [Pseudonocardia dioxanivorans CB1190]	AEA28277	Grostern	CB1190	upregulated with dioxane AND glycolate		
53	Psed_6779 | Integrase catalytic region (plasmid) [Pseudonocardia dioxanivorans CB1190]	AEA28859	Grostern	CB1190	upregulated with dioxane AND glycolate	K07497	K07497; putative transposase
54	Psed_6784 | Alkylglycerone-phosphate synthase (plasmid) [Pseudonocardia dioxanivorans CB1190]	AEA28862	Grostern	CB1190	upregulated with dioxane AND glycolate	K00803	AGPS, agpS; alkyldihydroxyacetonephosphate synthase [EC:2.5.1.26]
55	Psed_6787 | Formyl-CoA transferase (plasmid) [Pseudonocardia dioxanivorans CB1190]	AEA28864	Grostern	CB1190	upregulated with dioxane AND glycolate	K01041	gcdG, caiB; glutaryl-CoA transferase [EC:2.8.3.-]
56	Psed_6791 | IstB domain protein ATP-binding protein (plasmid) [Pseudonocardia dioxanivorans CB1190]	AEA28867	Grostern	CB1190	upregulated with dioxane AND glycolate		

	S1			S2			S3			S4			S5			S6			
	# hits	min % ID	max % ID	# hits	min % ID	max % ID	# hits	min % ID	max % ID	# hits	min % ID	max % ID	# hits	min % ID	max % ID	# hits	min % ID	max % ID	
1	0	N/A	N/A	0	N/A	N/A	0	N/A	N/A	0	N/A	N/A	0	N/A	N/A	0	N/A	N/A	1
2	4	47.388	89.003	4	47.388	89.474	6	47.388	93.373	4	47.015	89.347	4	47.036	90.378	3	47.388	89.691	2
3	12	56.915	78.692	14	56.915	78.692	18	56.915	80.769	17	56.915	81.818	13	56.915	78.692	18	56.915	79.641	3
4	186	26.847	99.306	192	26.847	97.018	190	28.51	96.907	227	26.847	97.018	246	26.847	97.018	201	26.847	96.789	4
5	10	51.299	86.709	8	51.299	89.474	9	51.299	89.51	9	51.299	86.709	10	51.299	87.342	11	51.299	87.342	5
6	71	32.199	84.401	92	31.937	84.456	76	32.021	88.068	86	31.937	85.714	78	31.937	85.047	77	31.937	89.32	6
7	5	39.221	83.92	5	39.221	84.383	4	39.221	84.171	3	39.221	83.92	4	39.481	84.131	4	39.221	87.342	7
8	2	85.577	91.589	2	85.577	90.909	2	85.577	91.346	2	84.135	85.577	3	49.758	85.577	2	85.577	92.929	8
9	304	30.144	93.293	354	30.144	89.56	322	30.1	93.642	412	30.212	88.535	449	29.846	93.431	339	30.144	86.992	9
10	176	35.727	87.19	223	35.175	87.19	192	35.727	87.19	222	35	87.19	258	34.868	87.19	215	35	87.19	10
11	0	N/A	N/A	0	N/A	N/A	0	N/A	N/A	0	N/A	N/A	0	N/A	N/A	0	N/A	N/A	11
12	280	35.463	93.491	316	35.463	93.919	276	35.463	93.204	283	38.692	91.008	322	35.463	91.214	277	35.463	94.054	12
13	0	N/A	N/A	1	40.845	40.845	0	N/A	N/A	0	N/A	N/A	0	N/A	N/A	0	N/A	N/A	13
14	1	88.043	88.043	2	88.043	88.172	2	88.043	88.172	1	88.043	88.043	1	88.043	88.043	2	88.043	89.655	14
15	186	31.278	56.287	222	32.08	57.021	193	31.323	53.429	240	31.278	54.124	287	30.769	61.333	246	31.278	57.895	15
16	0	N/A	N/A	0	N/A	N/A	0	N/A	N/A	0	N/A	N/A	0	N/A	N/A	0	N/A	N/A	16
17	0	N/A	N/A	0	N/A	N/A	0	N/A	N/A	0	N/A	N/A	0	N/A	N/A	0	N/A	N/A	17
18	0	N/A	N/A	0	N/A	N/A	0	N/A	N/A	0	N/A	N/A	0	N/A	N/A	0	N/A	N/A	18
19	227	30.844	98.561	285	30.757	96.748	239	30.4	94.6	242	31.357	95.395	258	31.086	95.595	236	31.086	98.802	19
20	152	29.472	53.149	170	29.472	53.149	163	29.472	52.897	191	29.472	63.087	197	29.472	57.075	180	29.472	52.897	20
21	32	36.471	65.318	39	36.39	65.318	37	35.955	65.318	38	33.811	65.318	38	36.389	65.318	35	36.389	65.318	21
22	17	51.744	63.699	26	50.943	65.323	19	51.744	63.699	29	51.744	63.699	28	51.744	63.699	26	51.705	63.699	22
23	7	46.535	53.769	9	49.231	56.213	8	48.718	53.769	6	50	53.769	8	44.898	53.769	6	50	53.769	23
24	12	50.568	94.304	13	37.919	92.544	10	50.568	95.652	13	50.568	95.683	17	50.568	96.703	14	50.568	97.143	24
25	33	42.416	100	30	43.434	100	38	43	100	34	44.966	94.894	42	42.405	100	31	43.434	95.753	25
26	6	37.059	62.278	9	37.214	75.145	10	37.214	78.344	6	37.214	64.244	9	37.087	78.806	9	37.214	78.788	26
27	21	38.439	82.836	24	38.574	84.076	21	38.574	83.162	24	38.574	88.542	26	38.574	83.162	22	38.574	88.542	27
28	145	26.981	97.149	165	26.981	100	160	26.981	99.495	170	26.78	97.674	183	26.72	100	171	26.981	100	28
29	10	56.306	99.065	8	63.866	100	7	63.866	98.354	10	63.866	98.354	10	63.866	98.354	8	63.866	98.354	29
30	4	80.392	99.065	4	81.132	99.065	4	81.132	99.065	4	81.132	99.065	3	81.132	99.065	3	81.132	99.065	30
31	56	33.813	100	63	33.813	98.99	53	34.709	100	66	34.509	97.073	65	33.813	97.073	61	34.509	97.073	31
32	56	33.813	100	63	33.813	98.99	53	34.709	100	66	34.509	97.073	65	33.813	97.073	61	34.509	97.073	32
33	14	39.319	85.143	21	39.319	86.525	17	39.319	84.494	15	38.413	84.862	18	38.291	85.053	16	39.319	84.494	33
34	0	N/A	N/A	0	N/A	N/A	0	N/A	N/A	0	N/A	N/A	0	N/A	N/A	0	N/A	N/A	34
35	0	N/A	N/A	0	N/A	N/A	0	N/A	N/A	0	N/A	N/A	0	N/A	N/A	0	N/A	N/A	35
36	500	30.632	61.331	500	32.533	61.364	500	30.632	61.702	500	34.563	61.364	500	34.25	61.506	500	33.019	68.045	36
37	16	35.476	92.982	17	37.049	92.982	15	35.476	96.739	13	45.598	92.982	12	35.476	92.982	12	36.991	92.982	37
38	116	33.414	88.406	152	31.63	89.455	131	33.987	88.406	144	33.414	93.373	128	33.247	87.302	128	33.414	87.302	38
39	11	29.762	85.294	18	29.282	82.883	13	29.282	81.481	9	28.814	72.043	9	33.918	79.592	10	33.918	75.652	39
40	3	77.391	92.373	3	79.13	92.373	2	91.525	92.373	2	91.525	92.373	2	91.525	93.333	2	91.525	92.373	40
41	16	45.148	80.657	14	45.082	80.657	11	47.148	81.022	14	45.082	80.657	13	45.082	80.657	13	45.082	80.657	41
42	114	35.54	87.458	143	35.54	87.458	119	35.54	87.458	133	36.332	87.458	127	36.897	87.458	127	35.54	87.458	42
43	98	35.833	94.444	113	35.837	95.27	95	39.744	94.444	125	39.712	94.239	140	35.897	94.041	124	36.474	94.444	43
44	15	38.014	91.333	18	38.014	98.113	16	37.671	91.333	15	38.014	91.333	14	37.671	91.333	12	38.014	91.333	44
45	2	79.293	80.357	3	76.037	81.223	1	79.5	79.5	2	75.814	76.389	1	76.037	76.037	3	75.814	77.483	45
46	0	N/A	N/A	0	N/A	N/A	0	N/A	N/A	0	N/A	N/A	0	N/A	N/A	0	N/A	N/A	46
47	265	31.685	92.14	325	31.685	95.062	293	31.685	92.14	317	31.685	92.14	336	31.685	92.14	320	31.685	92.14	47
48	161	30.152	88.421	172	30.152	88.421	179	30.152	94.545	206	30.019	88.421	222	30.019	88.421	187	30.152	88.421	48
49	7	64.824	93.596	6	64.824	93.103	7	64.824	95.276	5	64.824	93.103	5	64.824	93.596	5	64.824	93.596	49
50	194	35.588	87.24	223	35.588	87.097	206	39.017	87.24	254	39.017	87.24	263	39.017	87.24	232	39.017	87.24	50
51	6	47.442	84.916	7	47.442	84.916	5	47.442	84.916	7	47.442	84.916	6	47.442	84.916	6	47.442	84.653	51
52	4	62.927	92.857	4	62.927	92.857	4	63.277	92.857	4	62.927	92.857	3	87.143	92.381	3	62.927	92.857	52
53	18	36.042	79.47	25	36.042	79.47	26	36.042	79.47	30	36.042	79.47	31	37.153	79.47	27	36.042	79.47	53
54	68	28.974	81.818	76	28.12	81.818	72	28.12	81.818	73	28.12	81.818	74	28.12	81.818	68	29.965	81.818	54
55	500	33.596	73.925	500	33.516	73.925	500	33.784	73.925	500	34.247	73.925	501	34.247	73.925	500	33.795	73.925	55
56	7	58.333	75.342	9	49.254	79.787	8	58.333	75.342	11	45.192	78.667	11	44.712	78.102	9	59.804	76.961	56

Table S6. Summary of BLASTp hits results for sequences that were upregulated in Pseudonocardia dioxanivorans CB1190 during dioxane and glycolate degradation in Lowry support media (2-4). Red rows indicate queries that had no hits in the Lowry samples. Blue rows and blue values indicate amino acid identities of 90+%.

	Query (sequences upregulated in CB1190 during dioxane and glycolate degradation)	Accession	Paper	Strain	Function	KO	Definition
1	Psed_0038 | regulatory protein ArsR [Pseudonocardia dioxanivorans CB1190]	AEA22320	Grostern	CB1190	upregulated with dioxane		
2	Psed_1076 | short-chain dehydrogenase/reductase SDR [Pseudonocardia dioxanivorans CB1190]	AEA23327	Grostern	CB1190	upregulated with dioxane		
3	Psed_1594 | protein of unknown function UPF0016 [Pseudonocardia dioxanivorans CB1190]	AEA23831	Grostern	CB1190	upregulated with dioxane	K23541	TMEM165, GDT1; Ca2+/H+ antiporter, TMEM165/GDT1 family
4	Psed_1658 | Trimethylamine-N-oxide reductase (cytochrome c) [Pseudonocardia dioxanivorans CB1190]	AEA23894	Grostern	CB1190	upregulated with dioxane		
5	Psed_3041 | NADH dehydrogenase (ubiquinone) 24 kDa subunit [Pseudonocardia dioxanivorans CB1190]	AEA25240	Grostern	CB1190	upregulated with dioxane	K00127	fdoI, fdsG; formate dehydrogenase subunit gamma
6	Psed_3564 | putative transcriptional regulator, GntR family [Pseudonocardia dioxanivorans CB1190]	AEA25736	Grostern	CB1190	upregulated with dioxane	K05825	LYSN; 2-aminoadipate transaminase [EC:2.6.1.-]
7	Psed_3934 | major facilitator superfamily MFS_1 [Pseudonocardia dioxanivorans CB1190]	AEA26101	Grostern	CB1190	upregulated with dioxane		
8	Psed_3935 | transcriptional regulator, GntR family [Pseudonocardia dioxanivorans CB1190]	AEA26102	Grostern	CB1190	upregulated with dioxane		
9	Psed_4146 | Potassium-transporting ATPase B chain [Pseudonocardia dioxanivorans CB1190]	AEA26309	Grostern	CB1190	upregulated with dioxane	K01547	kdpB; potassium-transporting ATPase ATP-binding subunit [EC:7.2.2.6]
10	Psed_4147 | Potassium-transporting ATPase A chain [Pseudonocardia dioxanivorans CB1190]	AEA26310	Grostern	CB1190	upregulated with dioxane	K01546	kdpA; potassium-transporting ATPase potassium-binding subunit
11	Psed_4148 | K+-transporting ATPase, F subunit [Pseudonocardia dioxanivorans CB1190]	AEA26311	Grostern	CB1190	upregulated with dioxane		
12	Psed_5135 | malic protein NAD-binding protein [Pseudonocardia dioxanivorans CB1190]	AEA27272	Grostern	CB1190	upregulated with dioxane	K00027	ME2, sfcA, maeA; malate dehydrogenase (oxaloacetate-decarboxylating) [EC:1.1.1.38]
13	Psed_5524 | hydrolase [Pseudonocardia dioxanivorans CB1190]	AEA27654	Grostern	CB1190	upregulated with dioxane		
14	Psed_5938 | glycoside hydrolase family 13 domain-containing protein [Pseudonocardia dioxanivorans CB1190]	AEA28060	Grostern	CB1190	upregulated with dioxane	K00700	GBE1, glgB; 1,4-alpha-glucan branching enzyme [EC:2.4.1.18]
15	Psed_6259 | FMN-dependent oxidoreductase, nitrilotriacetate monooxygenase family [Pseudonocardia dioxanivorans CB1190]	AEA28360	Grostern	CB1190	upregulated with dioxane	K20938	ladA; long-chain alkane monooxygenase [EC:1.14.14.28]
16	Psed_6261 | ABC-type transporter, integral membrane subunit [Pseudonocardia dioxanivorans CB1190]	AEA28362	Grostern	CB1190	upregulated with dioxane	K01998	livM; branched-chain amino acid transport system permease protein
17	Psed_6262 | ABC-type transporter, integral membrane subunit [Pseudonocardia dioxanivorans CB1190]	AEA28363	Grostern	CB1190	upregulated with dioxane	K01997	livH; branched-chain amino acid transport system permease protein
18	Psed_6263 | Extracellular ligand-binding receptor [Pseudonocardia dioxanivorans CB1190]	AEA28364	Grostern	CB1190	upregulated with dioxane	K01999	livK; branched-chain amino acid transport system substrate-binding protein
19	Psed_6600 | acyl-CoA dehydrogenase domain-containing protein [Pseudonocardia dioxanivorans CB1190]	AEA28688	Grostern	CB1190	upregulated with dioxane		
20	Psed_6728 | Amidase (plasmid) [Pseudonocardia dioxanivorans CB1190]	AEA28809	Grostern	CB1190	upregulated with dioxane	K02433	gatA, QRSL1; aspartyl-tRNA(Asn)/glutamyl-tRNA(Gln) amidotransferase subunit A [EC:6.3.5.6 6.3.
21	Psed_6730 | Luciferase-like, subgroup (plasmid) [Pseudonocardia dioxanivorans CB1190]	AEA28811	Grostern	CB1190	upregulated with dioxane	K09018	rutA; pyrimidine oxygenase [EC:1.14.99.46]
22	Psed_6732 | MaoC domain protein dehydratase (plasmid) [Pseudonocardia dioxanivorans CB1190]	AEA28813	Grostern	CB1190	upregulated with dioxane	K18290	ich-Y; itaconyl-CoA hydratase [EC:4.2.1.56]
23	Psed_6735 | flavoprotein WrbA (plasmid) [Pseudonocardia dioxanivorans CB1190]	AEA28816	Grostern	CB1190	upregulated with dioxane	K03809	wrbA; NAD(P)H dehydrogenase (quinone) [EC:1.6.5.2]
24	Psed_6742 | NLP/P60 protein (plasmid) [Pseudonocardia dioxanivorans CB1190]	AEA28823	Grostern	CB1190	upregulated with dioxane		
25	Psed_6745 | ATP-binding protein (plasmid) [Pseudonocardia dioxanivorans CB1190]	AEA28826	Grostern	CB1190	upregulated with dioxane		
26	Psed_6751 | Transglycosylase-like domain protein (plasmid) [Pseudonocardia dioxanivorans CB1190]	AEA28832	Grostern	CB1190	upregulated with dioxane	K21687	rpfA; resuscitation-promoting factor RpfA
27	Psed_6799 | transposase IS4 family protein (plasmid) [Pseudonocardia dioxanivorans CB1190]	AEA28871	Grostern	CB1190	upregulated with dioxane		
28	Psed_6970 | D-lactate dehydrogenase (cytochrome) (plasmid) [Pseudonocardia dioxanivorans CB1190]	AEA29032	Grostern	CB1190	upregulated with dioxane		
29	Psed_6972 | transcriptional regulator, GntR family (plasmid) [Pseudonocardia dioxanivorans CB1190]	AEA29034	Grostern	CB1190	upregulated with dioxane		
30	Psed_6974 | Ethyl tert-butyl ether degradation EthD (plasmid) [Pseudonocardia dioxanivorans CB1190]	AEA29036	Grostern	CB1190	upregulated with dioxane		
31	Psed_6982 | Mn2+/Fe2+ transporter, NRAMP family (plasmid) [Pseudonocardia dioxanivorans CB1190]	AEA29043	Grostern	CB1190	upregulated with dioxane	K03322	mntH; manganese transport protein
32	Psed_7002 | Mn2+/Fe2+ transporter, NRAMP family (plasmid) [Pseudonocardia dioxanivorans CB1190]	AEA29059	Grostern	CB1190	upregulated with dioxane	K03322	mntH; manganese transport protein
33	Psed_0350 | Bile acid:sodium symporter [Pseudonocardia dioxanivorans CB1190]	AEA22623	Grostern	CB1190	upregulated with dioxane AND glycolate	K14347	SLC10A7, P7; solute carrier family 10 (sodium/bile acid cotransporter), member 7
34	Psed_1302 | protein of unknown function DUF156 [Pseudonocardia dioxanivorans CB1190]	AEA23545	Grostern	CB1190	upregulated with dioxane AND glycolate	K21600	csoR, ricR; CsoR family transcriptional regulator, copper-sensing transcriptional repressor
35	Psed_1303 | Heavy metal transport/detoxification protein [Pseudonocardia dioxanivorans CB1190]	AEA23546	Grostern	CB1190	upregulated with dioxane AND glycolate		
36	Psed_1304 | heavy metal translocating P-type ATPase [Pseudonocardia dioxanivorans CB1190]	AEA23547	Grostern	CB1190	upregulated with dioxane AND glycolate	K17686	copA, ctpA, ATP7; P-type Cu+ transporter [EC:7.2.2.8]
37	Psed_1584 | 6-phosphofructokinase [Pseudonocardia dioxanivorans CB1190]	AEA23821	Grostern	CB1190	upregulated with dioxane AND glycolate	K00850	pfkA, PFK; 6-phosphofructokinase 1 [EC:2.7.1.11]
38	Psed_2030 | Linalool 8-monooxygenase [Pseudonocardia dioxanivorans CB1190]	AEA24257	Grostern	CB1190	upregulated with dioxane AND glycolate		
39	Psed_2371 | ABC-type transporter, periplasmic subunit [Pseudonocardia dioxanivorans CB1190]	AEA24577	Grostern	CB1190	upregulated with dioxane AND glycolate	K02035	ABC.PE.S; peptide/nickel transport system substrate-binding protein
40	Psed_3522 | response regulator receiver protein [Pseudonocardia dioxanivorans CB1190]	AEA25695	Grostern	CB1190	upregulated with dioxane AND glycolate		
41	Psed_3888 | Hydroxypyruvate isomerase [Pseudonocardia dioxanivorans CB1190]	AEA26056	Grostern	CB1190	upregulated with dioxane AND glycolate	K01816	hyi, gip; hydroxypyruvate isomerase [EC:5.3.1.22]
42	Psed_3889 | 2-hydroxy-3-oxopropionate reductase [Pseudonocardia dioxanivorans CB1190]	AEA26057	Grostern	CB1190	upregulated with dioxane AND glycolate	K00042	garR, glxR; 2-hydroxy-3-oxopropionate reductase [EC:1.1.1.60]
43	Psed_4031 | iron-sulfur cluster binding protein [Pseudonocardia dioxanivorans CB1190]	AEA26196	Grostern	CB1190	upregulated with dioxane AND glycolate	K18929	lldF; L-lactate dehydrogenase complex protein LldF
44	Psed_4512 | ABC-type transporter, integral membrane subunit [Pseudonocardia dioxanivorans CB1190]	AEA26667	Grostern	CB1190	upregulated with dioxane AND glycolate	K15554	ssuC; sulfonate transport system permease protein
45	Psed_4513 | cobalamin (vitamin B12) biosynthesis CbiX protein [Pseudonocardia dioxanivorans CB1190]	AEA26668	Grostern	CB1190	upregulated with dioxane AND glycolate		
46	Psed_4577 | Cold-shock protein DNA-binding protein [Pseudonocardia dioxanivorans CB1190]	AEA26729	Grostern	CB1190	upregulated with dioxane AND glycolate		
47	Psed_4755 | ammonium transporter [Pseudonocardia dioxanivorans CB1190]	AEA26902	Grostern	CB1190	upregulated with dioxane AND glycolate	K03320	amt, AMT, MEP; ammonium transporter, Amt family
48	Psed_5025 | 4-hydroxyacetophenone monooxygenase [Pseudonocardia dioxanivorans CB1190]	AEA27163	Grostern	CB1190	upregulated with dioxane AND glycolate		
49	Psed_5026 | regulatory protein TetR [Pseudonocardia dioxanivorans CB1190]	AEA27164	Grostern	CB1190	upregulated with dioxane AND glycolate		
50	Psed_5406 | NAD(P)(+) transhydrogenase (AB-specific) [Pseudonocardia dioxanivorans CB1190]	AEA27539	Grostern	CB1190	upregulated with dioxane AND glycolate	K00324	pntA; proton-translocating NAD(P)+ transhydrogenase subunit alpha [EC:7.1.1.1]
51	Psed_5736 | Transglycosylase-like domain protein [Pseudonocardia dioxanivorans CB1190]	AEA27863	Grostern	CB1190	upregulated with dioxane AND glycolate	K21687	rpfA; resuscitation-promoting factor RpfA
52	Psed_6175 | regulatory protein TetR [Pseudonocardia dioxanivorans CB1190]	AEA28277	Grostern	CB1190	upregulated with dioxane AND glycolate		
53	Psed_6779 | Integrase catalytic region (plasmid) [Pseudonocardia dioxanivorans CB1190]	AEA28859	Grostern	CB1190	upregulated with dioxane AND glycolate	K07497	K07497; putative transposase
54	Psed_6784 | Alkylglycerone-phosphate synthase (plasmid) [Pseudonocardia dioxanivorans CB1190]	AEA28862	Grostern	CB1190	upregulated with dioxane AND glycolate	K00803	AGPS, agpS; alkyldihydroxyacetonephosphate synthase [EC:2.5.1.26]
55	Psed_6787 | Formyl-CoA transferase (plasmid) [Pseudonocardia dioxanivorans CB1190]	AEA28864	Grostern	CB1190	upregulated with dioxane AND glycolate	K01041	gcdG, caiB; glutaryl-CoA transferase [EC:2.8.3.-]
56	Psed_6791 | IstB domain protein ATP-binding protein (plasmid) [Pseudonocardia dioxanivorans CB1190]	AEA28867	Grostern	CB1190	upregulated with dioxane AND glycolate		

S7			S8			S9			S10			S11			S12			
# hits	min % ID	max % ID	# hits	min % ID	max % ID	# hits	min % ID	max % ID	# hits	min % ID	max % ID	# hits	min % ID	max % ID	# hits	min % ID	max % ID	
0	N/A	N/A	0	N/A	N/A	0	N/A	N/A	0	N/A	N/A	0	N/A	N/A	0	N/A	N/A	1
3	47.388	89.003	2	88.502	89.105	5	47.388	91.667	4	47.388	89.691	7	47.388	93.373	3	47.388	89.347	2
12	56.915	78.692	16	56.915	78.692	22	56.915	78.692	16	56.915	78.692	19	56.915	81.651	15	56.915	78.692	3
215	26.847	99.333	208	26.847	97.661	245	27.131	97.018	238	26.847	99.401	280	26.847	97.018	247	27.131	97.018	4
8	51.299	86.709	9	51.299	90	13	51.299	93.617	10	51.299	86.709	13	51.299	92.391	10	51.299	90	5
83	31.937	85.876	64	31.937	83.42	91	31.937	85.311	79	31.937	84.768	96	31.937	84.456	90	31.937	90.604	6
4	39.221	83.92	4	39.481	88.43	5	39.221	83.879	5	39.221	85.938	6	39.221	86.025	3	39.221	83.92	7
3	76.271	93	2	49.758	85.577	3	79.73	91.429	3	49.758	91.667	4	80.132	93.878	3	49.758	93	8
404	30.357	90.574	391	30.144	88.199	420	30.144	85.926	410	30.212	96	442	30.212	93.75	423	29.889	89.189	9
222	34.532	87.19	229	35	87.19	252	35	86.777	248	35	87.19	277	34.753	87.845	257	35.42	87.19	10
0	N/A	N/A	0	N/A	N/A	0	N/A	N/A	0	N/A	N/A	0	N/A	N/A	0	N/A	N/A	11
294	35.463	94.054	293	35.463	90.439	328	35.463	93.913	323	35.463	94.054	343	35.669	90.439	335	36.076	93.407	12
0	N/A	N/A	0	N/A	N/A	0	N/A	N/A	0	N/A	N/A	0	N/A	N/A	0	N/A	N/A	13
1	88.043	88.043	2	88.043	90.805	2	87.097	88.043	1	88.043	88.043	3	88.043	90.805	1	88.043	88.043	14
258	31.278	56.463	257	31.278	55.333	294	31.266	56.391	276	31.278	60.638	311	31.278	58.553	286	31.278	54.589	15
0	N/A	N/A	0	N/A	N/A	0	N/A	N/A	0	N/A	N/A	0	N/A	N/A	0	N/A	N/A	16
0	N/A	N/A	0	N/A	N/A	0	N/A	N/A	0	N/A	N/A	0	N/A	N/A	0	N/A	N/A	17
0	N/A	N/A	0	N/A	N/A	0	N/A	N/A	0	N/A	N/A	0	N/A	N/A	0	N/A	N/A	18
242	31.414	94.6	243	30.885	96.167	280	30.099	98.802	257	31.086	94.704	310	31.086	98.802	271	31.086	97.436	19
185	29.472	59.551	176	29.472	53.719	222	29.472	53.149	195	29.472	53.149	227	29.472	53.149	212	29.472	57.542	20
38	36.389	66.116	33	36.389	65.318	41	33.333	65.318	43	33.333	65.318	47	33.811	65.318	45	36.389	77.778	21
27	51.235	63.699	25	51.744	63.699	27	51.744	63.699	31	51.744	63.699	32	51.744	77.099	31	50	63.699	22
9	50	54.211	7	49.231	53.769	8	46.667	53.769	10	45.856	54.211	11	45.161	55.33	10	44.898	54.211	23
11	50.568	93.801	14	61.039	97.297	19	50.568	95.918	15	50.568	95.07	17	50.568	93.443	16	50.568	97.727	24
39	43.434	100	31	42.913	98.947	38	41.525	100	34	44.944	96.875	40	42.216	95.634	36	43.434	100	25
8	37.214	61.682	6	38.374	65.698	9	37.214	78.182	9	37.214	77.193	10	37.214	84.091	7	37.214	75.145	26
27	38.574	84.076	24	38.574	82.639	24	38.574	88.542	27	38.574	83.436	27	38.574	83.162	29	38.574	83.162	27
172	26.928	100	179	26.981	100	199	26.84	98.958	186	26.467	96.718	214	26.981	98.368	202	26.919	99.174	28
7	63.866	98.354	7	54.978	98.354	9	63.866	98.636	7	63.866	98.354	9	60.119	98.99	9	63.866	98.354	29
3	81.132	99.065	3	81.132	99.065	4	81.132	99.065	3	80.392	99.065	3	81.132	99.065	3	80.392	99.065	30
58	34.509	96.829	52	34.509	97.073	67	34.509	97.073	62	34.509	96.852	82	34.509	97.073	71	34.509	98.148	31
58	34.509	96.829	52	34.509	97.073	67	34.509	97.073	62	34.509	96.852	82	34.509	97.073	71	34.509	98.148	32
14	38.413	84.494	17	39.557	87.432	21	38.413	85.581	15	38.413	84.494	21	38.413	87	18	38.413	84.494	33
0	N/A	N/A	0	N/A	N/A	0	N/A	N/A	0	N/A	N/A	0	N/A	N/A	0	N/A	N/A	34
0	N/A	N/A	0	N/A	N/A	0	N/A	N/A	0	N/A	N/A	0	N/A	N/A	0	N/A	N/A	35
500	34.563	63.75	500	33.242	61.517	500	35.831	65.562	500	34.606	63.818	500	36.74	65.562	500	35.363	61.506	36
13	37.536	92.982	14	35.476	92.982	13	35.476	92.982	10	46.761	92.982	21	35.833	92.982	12	47.209	92.982	37
145	32.915	87.302	128	33.247	87.302	155	33.414	91.525	142	33.247	88.026	170	33.247	91.358	151	33.247	87.302	38
10	33.918	78.512	9	34.105	75.926	9	31.034	70.312	9	33.918	71.552	12	32.087	77.876	10	32.087	70.312	39
2	90.678	92.373	2	91.525	92.373	2	93.103	93.103	2	91.525	92.308	2	91.525	92.373	2	91.525	92.373	40
14	45.082	80.657	13	45.082	80.657	15	45.082	80.657	16	45.082	80.657	14	45.082	80.657	16	45.082	80.657	41
138	35.54	87.458	122	35.932	88.489	155	36.332	87.458	147	36.823	87.119	154	37.063	87.458	158	35.64	87.458	42
130	39.744	94.239	123	39.744	94.118	145	38.952	94.033	131	37.818	95.918	158	38.243	94.444	142	37.669	94.239	43
15	38.014	91.333	13	37.586	91.333	16	39.583	95	15	38.014	91.333	18	38.014	91.333	18	38.014	91.975	44
2	76.037	80.5	1	77.5	77.5	2	77.5	84.783	2	71.658	76.037	2	75.814	76.389	2	77.5	77.866	45
0	N/A	N/A	0	N/A	N/A	0	N/A	N/A	0	N/A	N/A	0	N/A	N/A	0	N/A	N/A	46
321	31.685	92.14	326	31.685	92.14	372	31.685	92.517	356	31.685	92.14	384	31.685	92.14	355	31.685	92.14	47
209	30.152	88.793	203	30.369	88.421	242	29.935	88.632	217	30.152	88.421	261	30.019	88.421	230	30.019	88.421	48
5	64.824	93.596	5	64.824	93.035	6	64.824	93.103	5	64.824	93.596	7	64.824	95.714	6	64.824	93.596	49
237	39.017	87.24	236	39.017	87.24	273	39.017	89.583	273	39.017	87.24	293	39.017	87.24	282	39.017	87.24	50
6	47.442	84.916	4	47.442	84.577	6	47.442	84.916	6	47.368	84.916	7	47.442	84.916	6	47.442	84.916	51
3	61.951	92.857	4	62.927	91.453	4	62.927	92.857	3	62.927	92.857	5	62.927	92.857	4	62.927	92.857	52
28	36.042	79.47	27	36.042	79.47	31	36.879	80.8	31	36.042	79.47	33	37.234	79.47	32	37.175	79.47	53
77	27.963	81.818	69	28.12	81.818	80	28.12	81.818	81	28.12	81.818	96	28.12	81.818	92	28.12	81.818	54
500	34.247	73.925	500	33.795	73.925	501	34.247	73.925	502	34.247	73.925	500	34.247	73.925	500	34.247	73.925	55
9	60.135	76.961	10	58.042	76.961	11	57.143	76.961	11	44.776	83.761	11	59.314	83.333	10	59.804	76.961	56

Table S6. Summary of BLASTp hits results for sequences that were upregulated in Pseudonocardia dioxanivorans CB1190 during dioxane and glycolate degradation in Lowry support media (continued) (2-4). Red rows indicate queries that had no hits in the Lowry samples. Blue rows and blue values indicate amino acid identities of 90+%.

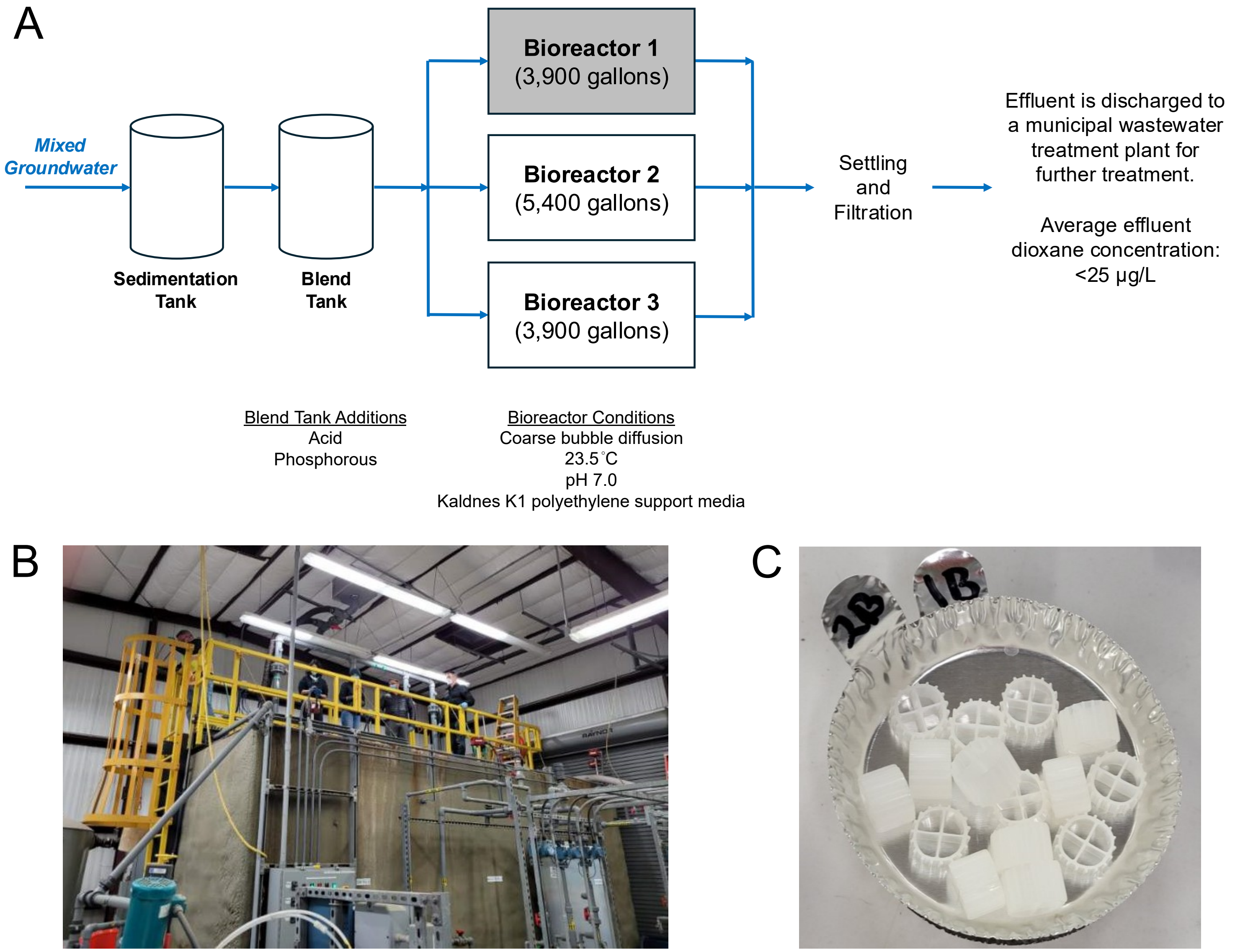

Fig S1. A schematic of the Lowry Landfill Biological Treatment System (BTS) (5) (A). Chemistry data was collected from the Sedimentation Tank. Support media from Bioreactor 1 (shaded) were sequenced. Photo of the bioreactors (B). Photo of uninoculated Kaldnes K1 polyethylene support media (C).

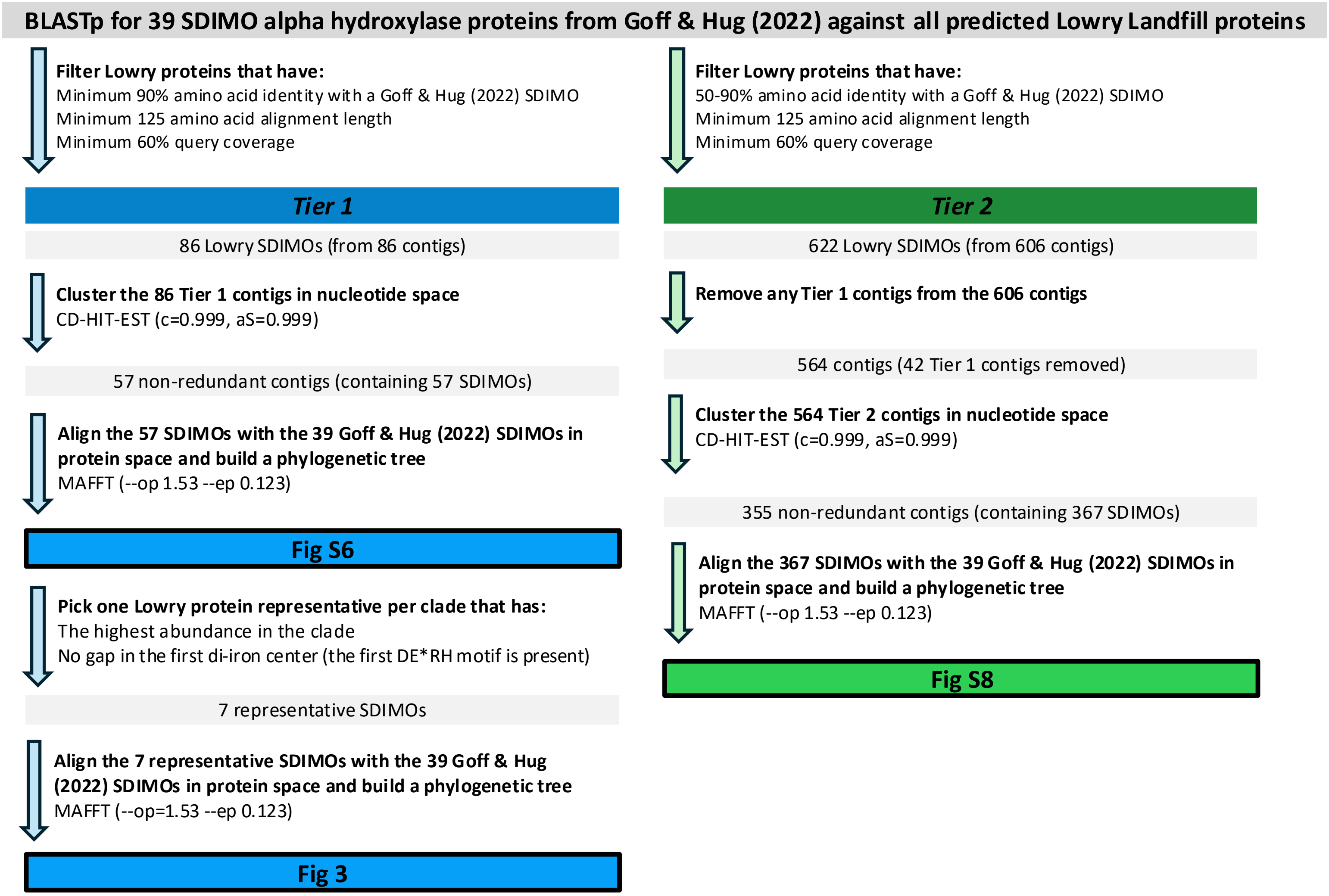

Fig S2. Flowchart of the tiered approach to rank SDIMO proteins curated from Lowry Landfill samples.

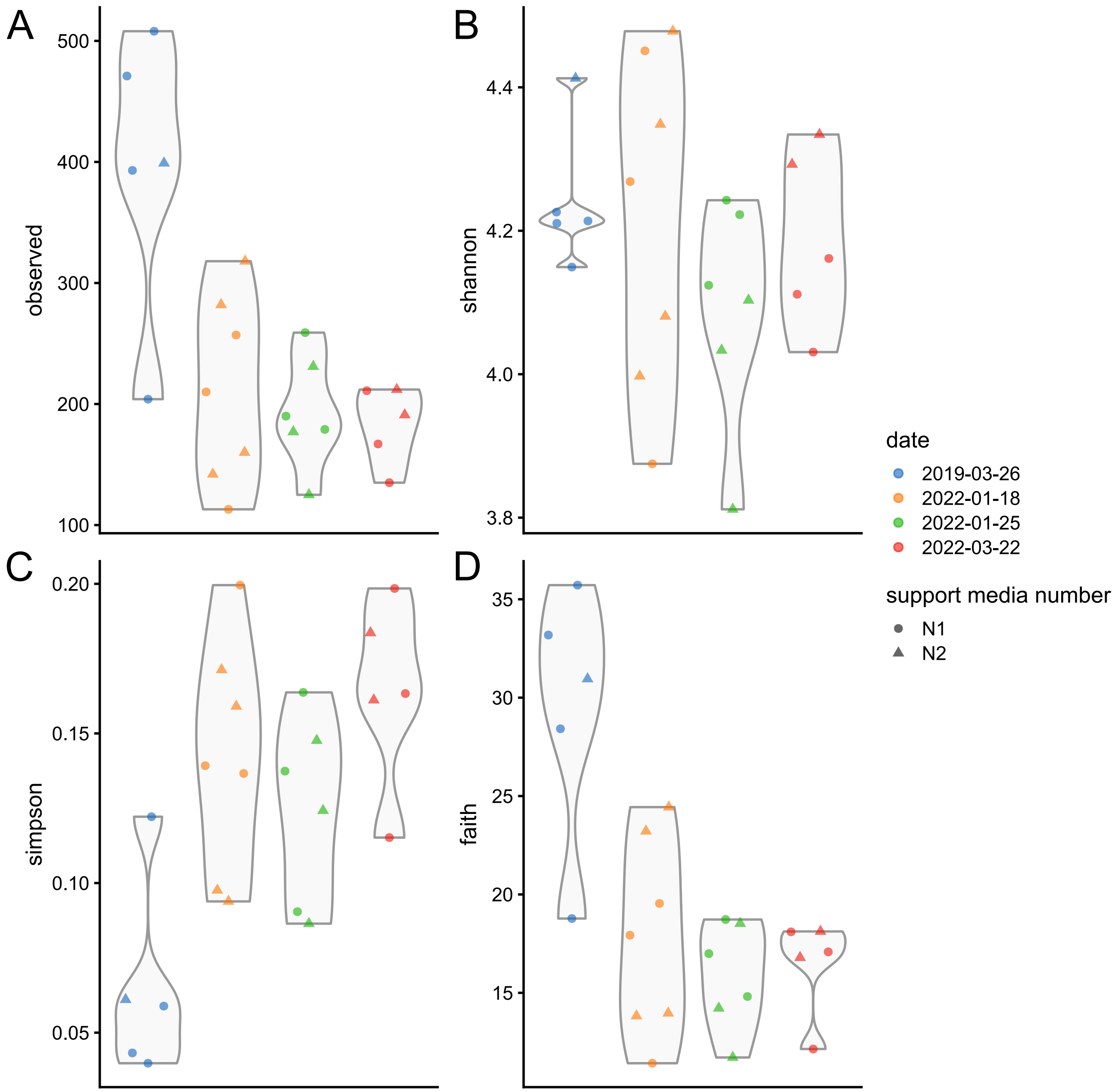

Fig S3. Alpha diversity analyses for Lowry Landfill Bioreactor 1 support media (n=23). The analyses performed included Observed Richness (A), Shannon (B), Simpson (C), and Faith PD (D) metrics.

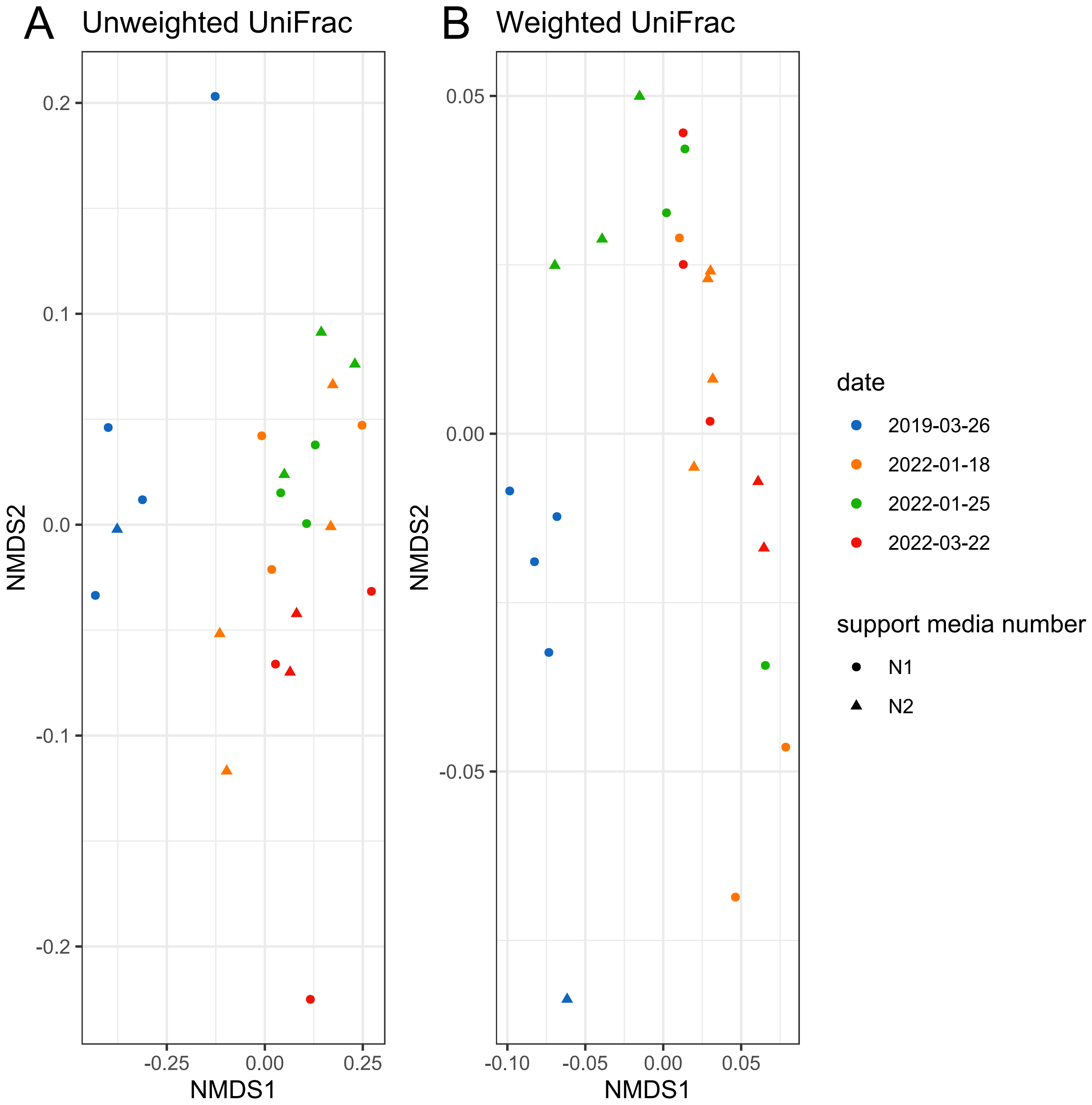

Fig S4. Beta diversity analyses for Lowry Landfill Bioreactor 1 support media (n=23). Unweighted (excluding taxon abundances, A) and weighted (including taxon abundances, B) non-metric multidimensional scaling (NMDS) analyses were based on UniFrac distances.

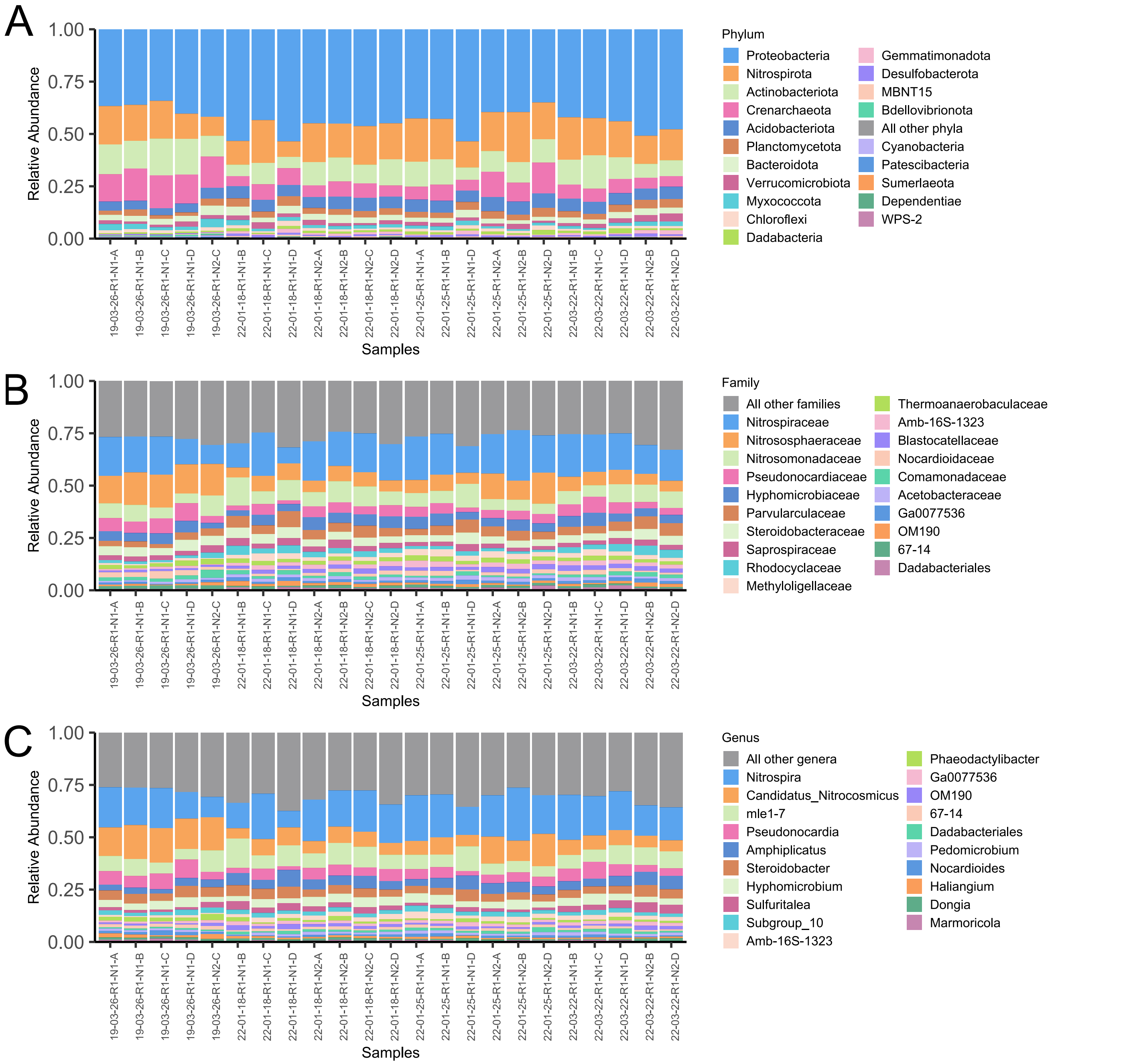

Fig S5. Microbial community composition bar plot of the top 20 most abundant phyla (A), families (B), and genera (C) in each Lowry Landfill Bioreactor 1 support media quarter (n=23). "All other phyla", "All other families", and "All other genera" represent taxa that were not among the top 20 most abundant taxa.

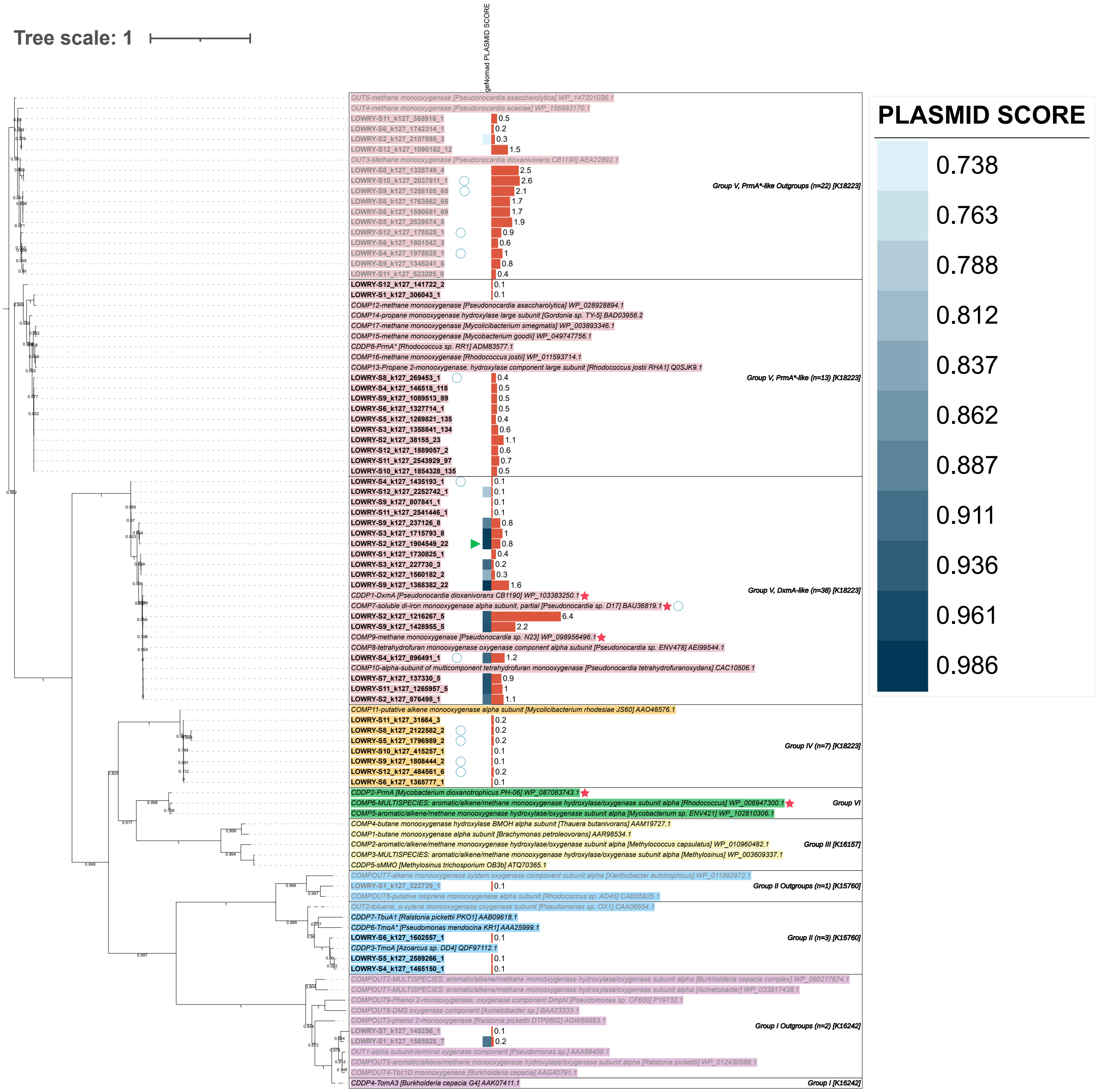

Fig S6. Protein phylogenetic tree of sequences recovered from Lowry Landfill Bioreactor 1 support media within 57 non-redundant contigs and 39 candidate SDIMOs described by K. L. Goff and L. A. Hug (1). Protein abundances were estimated using coverages (RPKM) of the 86 original Lowry contigs containing SDIMOs and displayed in bar charts for each sequence. Sequences containing an inverted terminal repeat according to geNomad are marked with a green triangle. Plasmid scores above the geNomad default threshold are displayed in the heatmap. See Fig. 3 legend for more details on symbols and labels.

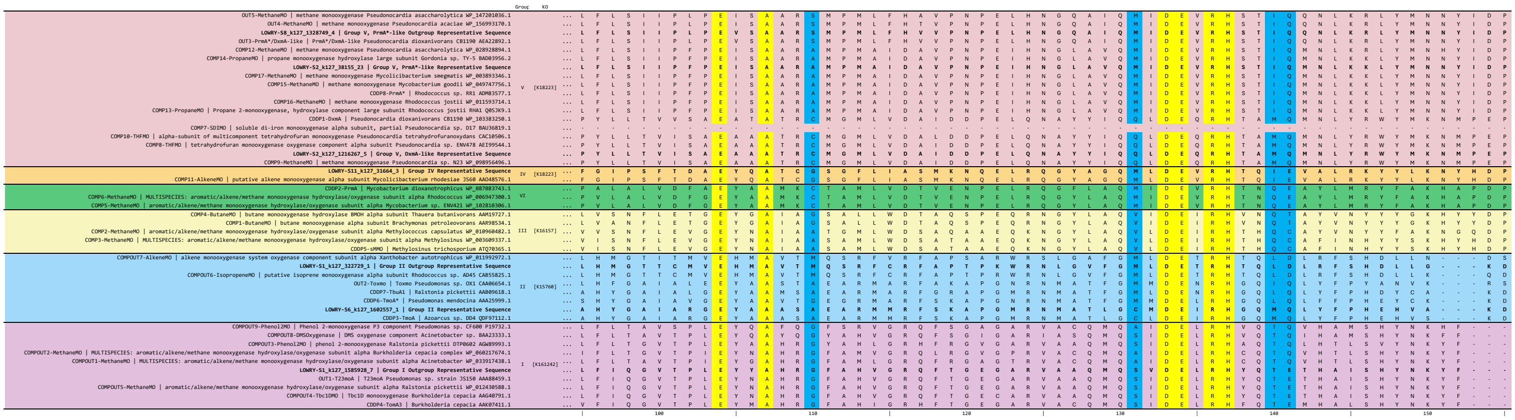

Fig S7. Amino acid alignment of the first region of the di-iron center of the alpha hydroxylase subunit of seven Lowry Landfill representative protein sequences and 39 candidate SDIMOs described by K. L. Goff and L. A. Hug (1) (positions 96-155). Residues highlighted in yellow showed high conservation. Residues highlighted in blue indicate hydrophobic amino acids used to classify sequences into SDIMO Groups I-VI.

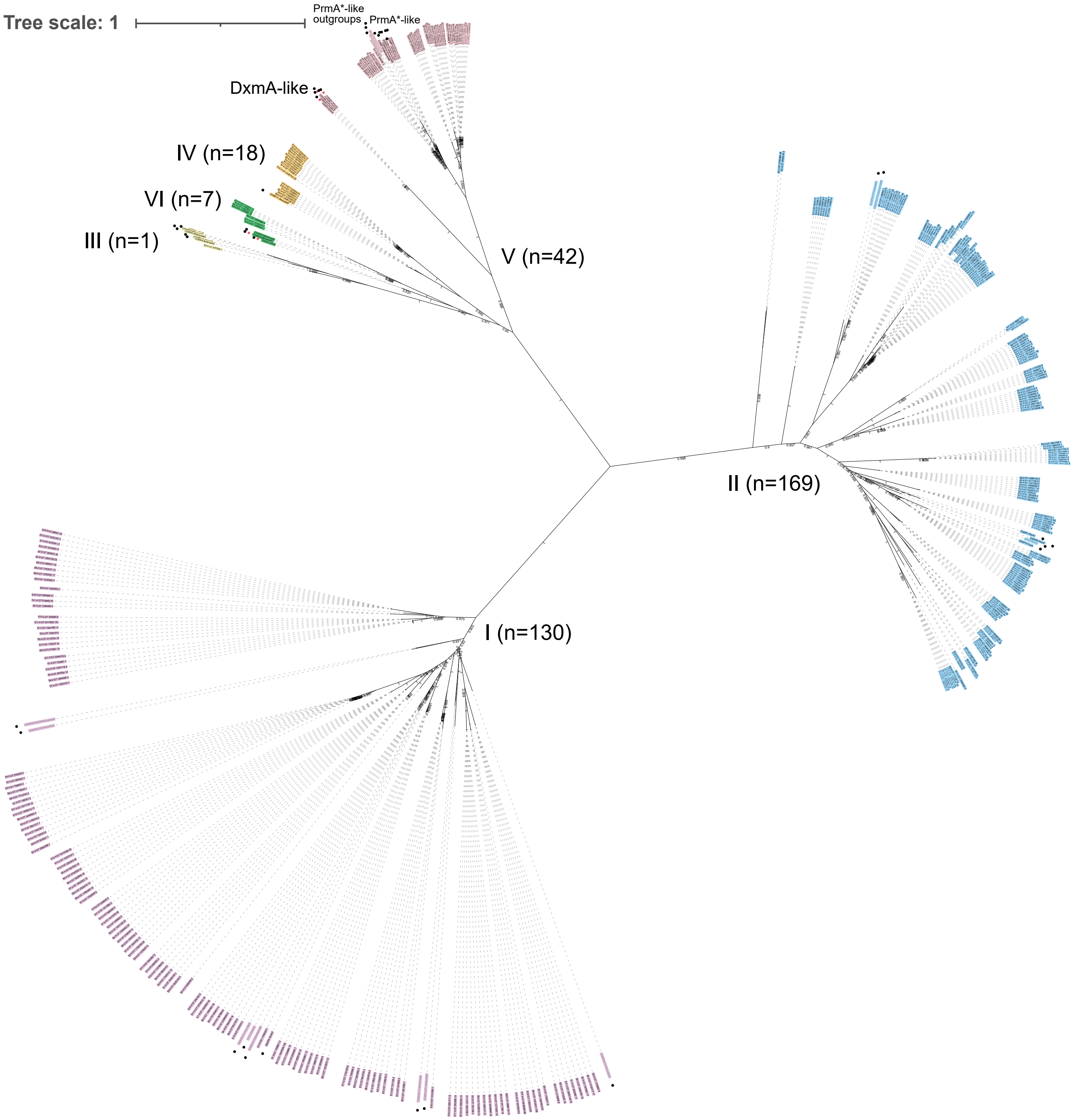

Fig S8. Unrooted protein phylogenetic tree of 367 Tier 2 potential SDIMO sequences recovered from Lowry Landfill Bioreactor 1 support media and 39 candidate SDIMOs described by K. L. Goff and L. A. Hug (1). The 367 potential SDIMO sequences were recovered from a set of 355 non-redundant contigs. Proteins from K. L. Goff and L. A. Hug (1) that are presumed not to degrade dioxane due to monophyletic clading with known outgroups are written in gray text. The branches for OUT2 and CDDP4 were dashed as these showed monophyletic clading that was unexpected for their description in K. L. Goff and L. A. Hug (1). Red stars indicate sequences that have shown direct metabolism of dioxane in the literature. Circles indicate sequences from K. L. Goff and L. A. Hug (1) whereas unmarked sequences were recovered from Lowry Landfill. Lowry sequences are colored by predicted SDIMO group based on clading patterns with literature sequences.

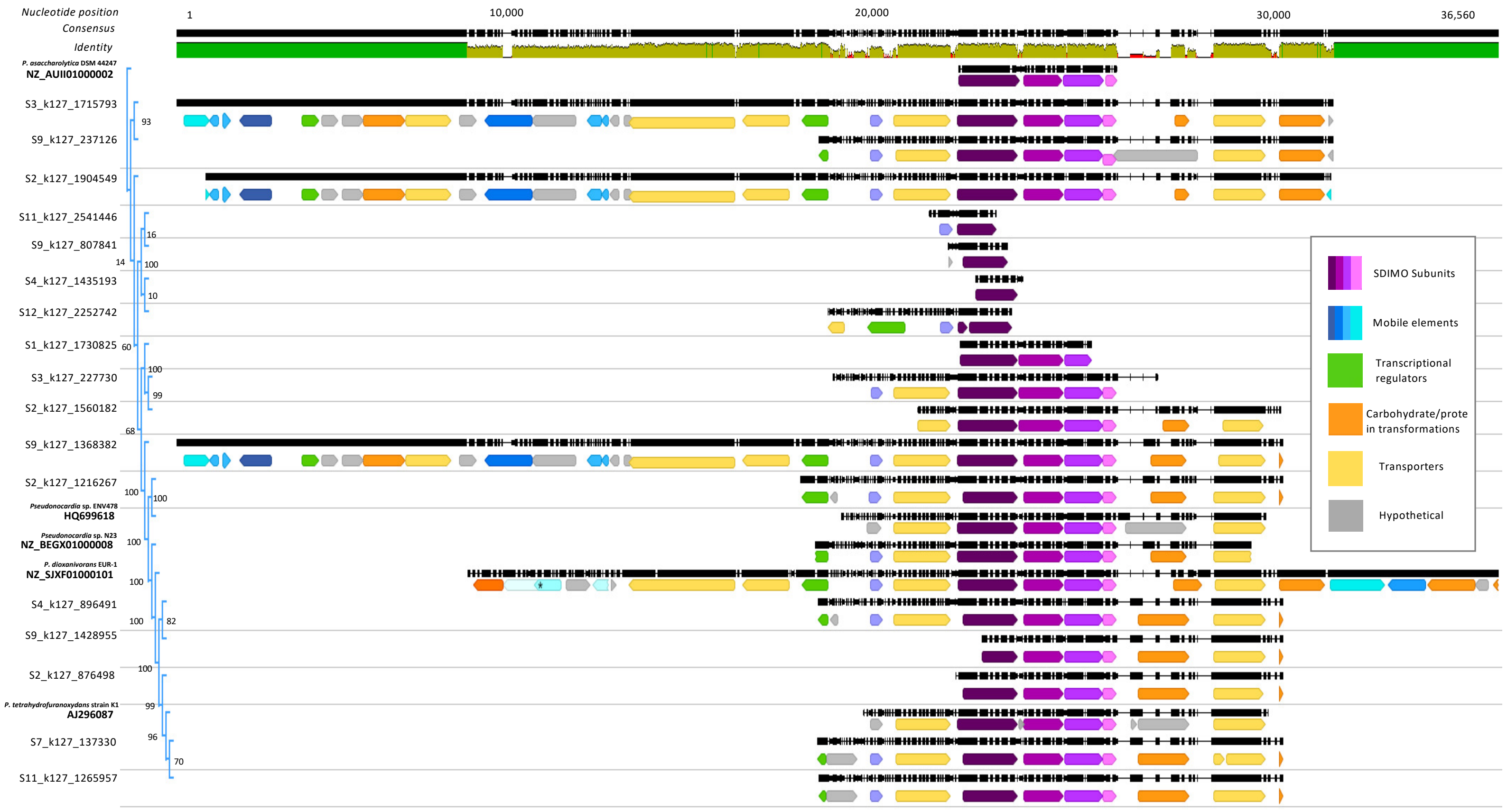

Fig S9. Alignment and phylogeny of the Lowry DxmA-like CDDP containing contigs, along with representative contigs from K. L. Goff and L. A. Hug (bolded) (1). Representative contigs include: *Pseudonocardia* sp. N23 (NZ_BEGX01000008), *Pseudonocardia* sp. ENV478 (HQ699618), *Pseudonocardia tetrahydrofuranoxydans* strain K1 (AJ296087*), Pseudonocardia dioxanivorans* EUR-1 (NZ_SJXF01000101), and the outgroup *Pseudonocardia asaccharolytica* DSM 44247 (NZ_AUII01000002). Predicted proteins and annotation categories are color coded by function (see inset). On the identity plot, green indicates 100% identity, while greenish brown indicates at least 30% and below 100%, and red indicates below 30%. Bootstrap values are denoted at the nodes of the phylogenetic tree.

Other Supplemental Materials

16S Supplemental Materials

ASV table with taxonomy from 16S rRNA sequencing data. Amplicon Sequence Variant (ASV) table with taxonomy strings (converted from the original BIOM-formatted table) derived from 16S rRNA sequencing of Lowry Bioreactor 1 support media. Read counts for all ASVs (n=884) across all samples (n=23) are provided.

Representative (ASV) sequences from 16S rRNA sequencing data. Representative (ASV) nucleotide sequences derived from 16S rRNA sequencing of Lowry Bioreactor 1 support media. ASV nucleotide sequences (n=884) are provided in FASTA format.

REFERENCES

1. Goff KL, Hug LA. 2022. Environmental Potential for Microbial 1,4-Dioxane Degradation Is Sparse despite Mobile Elements Playing a Role in Trait Distribution. Applied and Environmental Microbiology 88:e02091-21.
2. Grostern A, Sales CM, Zhuang WQ, Erbilgin O, Alvarez-Cohen L. 2012. Glyoxylate Metabolism Is a Key Feature of the Metabolic Degradation of 1,4-Dioxane by *Pseudonocardia dioxanivorans* Strain CB1190. Applied and Environmental Microbiology 78:3298-3308.
3. Chen RH, Miao Y, Liu Y, Zhang L, Zhong M, Adams JM, Dong YH, Mahendra S. 2021. Identification of novel 1,4-dioxane degraders and related genes from activated sludge by taxonomic and functional gene sequence analysis. Journal of Hazardous Materials 412.
4. Dai CH, Wu H, Wang XJ, Zhao KK, Lu ZM. 2022. Network and meta-omics reveal the cooperation patterns and mechanisms in an efficient 1,4-dioxane-degrading microbial consortium. Chemosphere 301.
5. Cordone L, Carlson C, Plaehn W, Shangraw T, Wilmoth D. 2016. Case Study and Retrospective: Aerobic Fixed Film Biological Treatment Process for 1,4-Dioxane at the Lowry Landfill Superfund Site. Remediation Journal 27:159-172.
